# CD121b-positive neutrophils predict immunosuppression in septic shock

**DOI:** 10.3389/fimmu.2025.1565797

**Published:** 2025-03-31

**Authors:** Jian Chen, Jinghe Zhang, Siao Chen, Jingkun Qin, Xinyu Hu, Shengdi Xi, Lin Zhang, Min Zhou, Yonggang Zhou, Binqing Fu, Haiming Wei

**Affiliations:** ^1^ Department of Critical Care Medicine, The First Affiliated Hospital of University of Science and Technology of China (USTC), Division of Life Science and Medicine, University of Science and Technology of China, Hefei, China; ^2^ The Key Laboratory of Immune Response and Immunotherapy, School of Basic Medical Sciences, Division of Life Sciences and Medicine, University of Science and Technology of China, Hefei, China; ^3^ Center for Advanced Interdisciplinary Science and Biomedicine of Institute of Health and Medicine (IHM), School of Basic Medical Sciences, Division of Life Sciences and Medicine, University of Science and Technology of China, Hefei, China; ^4^ Institute of Immunology, University of Science and Technology of China, Hefei, China

**Keywords:** septic shock, neutrophils, immunosuppression, CD121b, cytokine

## Abstract

**Background:**

Septic shock is linked with high mortality and significant long-term morbidity in survivors. However, the specific role of neutrophils in septic shock pathophysiology remains scarce in recent research.

**Methods:**

Peripheral blood immune cells from healthy donors and patients with septic shock were analyzed using single-cell RNA sequencing and batch RNA sequencing. We measured serum CD121b in both patients and healthy donors. Peripheral immune cells were isolated and exposed to either a CD121b recombinant protein or a CD121b blocking antibody to evaluate the expression of inflammatory factors. Additionally, in a humanized mouse sepsis model, the expression of CD121b in neutrophils across different tissues was assessed following treatment with all-trans retinoic acid (ATRA).

**Results:**

This study identified a subset of CD10^-^CD121b^+^ neutrophils in the peripheral blood of patients with septic shock. These patients exhibited elevated concentrations of soluble CD121b in serum and urine. Furthermore, outcomes revealed that the presence of CD121b^+^ neutrophils positively correlated with the severity of septic shock. These cells displayed immunosuppressive characteristics; after blocking CD121b, proinflammatory cytokines increased in peripheral immune cells. Additionally, we found that treatment with ATRA down-regulated the expression of CD121b.

**Conclusions:**

CD121b is closely associated with the progression of septic shock and may serve as a potential predictor indicator of immunosuppression for the condition.

## Introduction

Sepsis, a life-threatening condition, is marked by a dysregulated immune response that involves the simultaneous occurrence of hyperinflammation and immune suppression (1, 2). Septic shock, the utmost severe form of sepsis, remains a major global healthcare challenge, leading to significant morbidity and mortality (3–5). While initially underrecognized, severe immunosuppression in sepsis patients is now well-researched and linked to harmful outcomes, primarily due to an increased susceptibility to secondary hospital-acquired infections (6, 7).

Outcomes from immunomodulatory treatments have been underwhelming due to the difficulty in identifying when patients enter the immunosuppressive phase of sepsis. *Research gap:* there is an urgent need for new and effective biomarkers that can definitively diagnose sepsis-induced immunosuppression in clinical settings.

CD121b designates the recombinant protein for IL-1 receptor II (IL1R2), encoded by the IL1R2 (8). It is a vital component of the IL-1 receptor family. In contrast to other family members, IL-1R2 possesses a shorter cytoplasmic domain. It lacks the Toll/IL-1 receptor (TIR) domain, rendering it incapable of initiating signal transduction (9, 10). The IL-1R2 protein has two forms: a membrane-bound receptor and a soluble variant (11). Its primary mechanism of action as a decoy receptor involves competitively capturing IL-1 at the cell surface and binding IL-1 in the microenvironment, thereby effectively inhibiting IL-1 activity (12). Recent research has identified immature CD121b^+^ neutrophils in patients with sepsis (13). CD121b is a decoy receptor for IL-1, suggesting that the subpopulation of CD121b^+^neutrophils may have immunosuppressive effects (12, 14). However, researchers have not yet thoroughly investigated the impact and underlying mechanisms of CD121b^+^ neutrophils on sepsis progression. The present *research aimed* to assess the extent of CD121b expression in patients with septic shock and investigate its relationship with the severity of sepsis. Additionally, we examined whether targeting CD121b could effectively treat sepsis-induced immunosuppression. The *core hypotheses* of this study are as follows: *1)* CD121b^+^ neutrophils are significantly elevated in patients with septic shock compared to those with sepsis alone, *2)* The impact of CD121b^+^ neutrophils on the progression of sepsis is associated with immune suppression, *3)* All-trans retinoic acid (ATRA) could downregulate CD121b expression on neutrophils and can be utilized for alleviating sepsis-induced immunosuppression.

## Materials and methods

### Patient recruitment

From September 2021 to December 2023, every consecutive patient admitted to the Intensive Care Unit of the First Affiliated Hospital of the University of Science and Technology of China, Hefei, China, was enrolled. All patients (aged 18-90 years, sex not specified) met the clinical criteria defined by Third International Consensus Definitions for Sepsis and Septic Shock (Sepsis-3). The segregation criteria were defined as follows: *(1)* pregnancy or lactation, *(2)* advanced-stage cancer with cachexia, and *(3)* granulocytopenia. We collected blood samples from ICU-admitted patients (ICU-Ad) within the first 24 hours of ICU admission. We obtained samples from ICU survivors (ICU-Survivor) within 24 hours of ICU discharge, meanwhile collecting samples from patients in recovery (Recovery) within 24 hours of hospital discharge.

Moreover, blood samples from stabilized ICU patients (ICU-Hosp) were collected within 24 hours of circulatory stabilization. The control group comprised healthy donors (HDs) aged 18 to 90 years, with no sex specification. Entire patients and their legal representatives gave written informed consent. **Table 1** outlines the clinical data for patients suffering from sepsis and for HDs. The *SI Appendix*, **Supplementary Table S1**, shows detailed information on patients.

**Table 1:** Baseline characteristics of patients, stratified according to study group.

	Healthy donors (n=113)	Non-sepsis (n=37)	Septic non-shock (n=46)	Septic shock (n=127)
Age, years, mean(± SEM)	51.0 (± 1.3)	62.1 (± 2.5)	64.5 (± 2.0)	66.3 (± 1.1)
Sex				
Male	75 (66.4%)	20 (54.1%)	31 (67.4%)	89 (70.1%)
Female	38 (33.6%)	17 (45.9%)	15 (32.6%)	38 (29.9%)
Source of infection				
Pulmonary	/	/	39	51
Abdominal	/	/	5	41
Urinary	/	/	1	9
Blood	/	/	0	6
Other	/	/	1	20
APACHE II, median (IQR)	/	12.0 (8.0 to 16.5)	16.0 (12.0 to 22.0)	21.0 (17.0 to 26.0)
SOFA, median (IQR)	/	0.0 (0.0 to 3.0)	6.0 (4.0 to 8.0)	6.0 (4.0 to 9.0)
Lactate, mmol/L, median (IQR)	/	/	2.0 (1.3 to 3.1)	10.0 (5.0 to 16.0)
ICU LOS, days, median (IQR)	/	2.0 (1.0 to 4.0)	11.0 (7.0 to 20.3)	3.4 (2.5 to 5.7)

Data are presented as n (%); mean (± SEM) or median with IQR (25th to 75th percentiles). APACHE II, acute physiology and chronic health evaluation score;SOFA, Sequential Organ Failure Assessment; LOS, length of stay.

### Mice and treatment

Female NOD/ShiLtJGpt-Prkdc^em26Cd52^Il2rg^em26Cd22^/Gpt (NCG) mice (6–8 weeks; catalog number: T001475) were purchased from GemPharmatech (Nanjing, China) for the generation of humanized mouse models. We housed all mice under specific pathogen-free conditions. White blood cells (WBCs) (1×10^7^) from patients with septic shock were intravenously injected into NCG mice through the tail vein to create a humanized mouse model. We gave mice ATRA (ATRA treatment group; 20mg/kg) or the corresponding solvent (Vehicle group); 24 hours later, we collected the liver, lungs, and spleen of mice for flow cytometry.

### Ethics

The Medical Research Ethics Committee of the First Affiliated Hospital of the University of Science and Technology of China approved this study (2022KY Ethics number 158). The Ethics Committee of the University of Science and Technology of China (Beijing, China) approved all animal experimental procedures, and these were undertaken following the National Guidelines for Animal Usage in Research in China (USTCACUC25120122038).

### Blood sample collection, preparation

The authors collected peripheral blood samples from donors following ethical guidelines and under sterile conditions. Samples were centrifuged at 600 × g for 10 minutes at room temperature to separate serum. For red blood cell (RBC) lysis, 2 mL of RBC Lysis Buffer was added to each tube containing up to 100 μL of whole blood, followed by gentle vortexing. The samples were then incubated at room temperature, protected from light, for 10 minutes. After incubation, we centrifuged the cell suspension at 350 × g for 10 minutes and carefully aspirated the supernatant without disturbing the cell pellet. We resuspended the resulting cell pellet in phosphate-buffered saline (PBS) for subsequent experiments.

### Flow cytometry and fluorescence-activated cell sorting

Human WBCs or mouse mononuclear cells isolated from the liver, lungs, and spleen resuspended in 100 μL of PBS containing a pre-mixed antibody cocktail. Cells were incubated at 4°C for 30 minutes, followed by two washes with cold PBS. Flow cytometric analysis occurred on a FACSCelesta™ system (BD Biosciences, Franklin Lakes, NJ, USA). For FACS purification, cells were sorted on a FACS Aria™ III Cell Sorter (BD Biosciences) using 1× PBS as the sheath fluid. Live cells were first gated by excluding zombie dye-positive cells, then removing debris using forward scatter area (FSC-A) and side scatter area (SSC-A) parameters. We excluded cell doublets and aggregates using FSC-width (FSC-W) and SSC-width (SSC-W) parameters. Neutrophils were identified and isolated based on CD45^+^CD14^−^CD16^+^ expression.

### Enzyme-linked immunosorbent assays

Serum levels of IL-1β, IL-4, IL-8, IL-10, IL-1R2, and IL-1RA were quantified using ELISA following the manufacturer’s protocols. Briefly, serum samples were applied to pre-coated 96-well plates and incubated with detection antibodies for 60 minutes at room temperature. After three washes, we incubated the plates with streptavidin–horseradish peroxidase for 20 minutes at room temperature. We added 3,3′,5,5-Tetramethylbenzidine substrate solution and stopped the enzymatic reaction after 2–5 minutes. Absorbance was measured at 450 nm using a plate reader.

### RT-qPCR

We extracted total RNA using TsingZol Reagent (TSP401; Tsingke Biotech, Beijing, China) and reverse transcribed into complementary DNA (cDNA) using MonScript™ RTIII All-in-One Mix with dsDNase (MR05101M; Monad Laboratories, New York, NY, USA). We used gene-specific primers to amplify target genes and quantified relative gene expression using real-time quantitative PCR (RT-qPCR) with the SYBR Green Pre-mix Pro Taq HS qPCR Kit (AG11746; Accurate Biology, Salem, NC, USA). We used β-actin as the internal control for normalization. **Table 2** shows the primer sequences used for qRT-PCR.

**Table 2:** qRT-PCR primers used in this study, related to STAR Methods.

Targeted gene	NCBI Gene ID.	Primer sequence
Human		
*ACTB*	60	Forward: TTGCCGACAGGATGCAGAA
		Reverse: GCCGATCCACACGGAGTACTT
*IL1R2*	7850	Forward: ATGTTGCGCTTGTACGTGTTG
		Reverse: CCCGCTTGTAATGCCTCCC
*IL1B*	3553	Forward: ATGATGGCTTATTACAGTGGCAA
		Reverse: GTCGGAGATTCGTAGCTGGA
*IL6*	3569	Forward: ACTCACCTCTTCAGAACGAATTG
		Reverse: CCATCTTTGGAAGGTTCAGGTTG
*TNF*	7124	Forward: CCTCTCTCTAATCAGCCCTCTG
		Reverse: GAGGACCTGGGAGTAGATGAG
*IL8*	3576	Forward: TTTTGCCAAGGAGTGCTAAAG
		Reverse: AACCCTCTGCACCCAGTTTTC
*CD10*	4311	Forward: AGAAGAAACAGCGATGGACTCC
		Reverse: CATAGAGTGCGATCATTGTCACA

### Peripheral blood smears and Wright–Giemsa staining

A 50-μL aliquot of human peripheral blood was aspirated and placed at one end of a glass slide using a micropipette. A second glass slide (the spreader) was positioned at an approximate 30° angle and gently brought into contact with the blood droplet. We retracted the spreader slide to allow even distribution of the blood between the two slides, followed by a forward motion to create a uniform smear. Wright–Giemsa stain (0.2–0.6 mL) was then applied, covering the entire smear, and left for 1 minute. We added an equal volume of PBS to the stain, mixed it gently, and incubated it for 3 minutes. We rinsed the slides with water, air-dried them, and subsequently examined them under a microscope for morphological analysis.

### Bulk RNA sequence

We extracted total RNAs from human neutrophils using TRIzol™ Reagent (15596026; Invitrogen, Carlsbad, CA, USA). We carried out DNA digestion after RNA extraction using DNaseI. We determined RNA quality by measuring absorbance at an excitation wavelength of 260 nm and an emission wavelength of 280 nm with a spectrophotometer (Nanodrop™; Thermo Fisher Scientific, Waltham, MA, USA). We confirmed RNA integrity by electrophoresis using 1.5% agarose gels. We quantified qualified RNAs by Qubit3.0 with the Qubit™ RNA Broad Range Assay kit (Q10210; Life Technologies, Carlsbad, CA, USA). We used 2 μg of total RNAs to prepare RNA sequencing libraries using the KC-Digital™ Stranded mRNA Library Prep Kit (DR08502; Wuhan Seqhealth, Wuhan, China) for an Illumina (San Diego, CA, USA) platform following the manufacturer’s instructions. This kit eliminated duplication bias in PCR and sequencing steps using a unique molecular identifier (UMI) of eight random bases to label pre-amplified cDNA molecules. Library products corresponding to 200-500 bps were enriched, quantified, and sequenced on a DNBSEQ-T7 sequencer (MGI Tech, Beijing, China) with the PE150 model. We started by filtering the raw sequencing data, eliminating low-quality reads and reducing adaptor-sequence-contaminated reads. Clean reads were treated with in-house scripts to eliminate the duplication bias introduced in library preparation and sequencing. In brief, clean reads were first clustered according to UMI sequences (i.e., reads with the same UMI sequence were grouped into the same cluster). Reads in the same cluster were compared by pairwise alignment, and then reads with sequence identity >95% were extracted to a new sub-cluster. After generating all sub-clusters, we carried multiple sequence alignments to obtain one consensus sequence for each sub-cluster. After these steps, we eliminated any errors and biases introduced by PCR amplification or sequencing.

### Single-cell collection, construction and sequencing of the library

Single-cell suspensions (2×10^5^ cells/mL) with PBS were loaded onto a microwell chip using the Matrix^®^ Single Cell Processing System (Singleron, Nanjing, China). Barcoding beads were collected from the microwell chip, followed by reverse transcription of the mRNA captured by the barcoding beads to obtain cDNA and PCR amplification. Then, we fragmented the amplified cDNA, ligated it with sequencing adapters, and constructed scRNA-sequencing libraries according to the protocol of the GEXSCOPE^®^ Single Cell RNA Library Kit (Singleron). Donor libraries were diluted to 4 nM, pooled, and sequenced on the NovaSeq 6000 system (Illumina) with 150-bp paired-end reads. Raw reads from scRNA-sequencing were processed to generate gene expression matrices using the CeleScope v1.9.0 pipeline on the reference genome GRCh38 to generate expression matrix files for subsequent analyses.

### scRNA sequencing analyses and workflows

We imported expression matrix files into the R package (R Institute of Statistical Computing, Vienna, Austria) Seurat (*version 4.3.0 and version 4*) and the python-based toolkit scanpy (version 3.11.4 and version 1.9.3) for normalization, scaling, integration, Louvain clustering, dimensionality reduction, analysis of differential expression, and visualization. We considered cells with abnormal transcriptional complexity (<500 UMIs, >30,000 UMIs, or >25% of mitochondrial reads) as artefacts and removed them from subsequent analyses. Cell clusters were merged for protein marker-based annotation of major known immune-cell types at a broad level and kept at the clustering resolution of choice for acceptable annotation, in which neutrophil clusters were annotated manually by querying known markers (i.e., CSF3R, CD177, S100A8 and S100A9). We confirmed annotation results with SingleR (v.1.6.1) assignments.

For RNA velocity, a BAM file containing the reference genome GRCh38 (hg38) was used in the analysis with velocity v0.2.3 and scVelo v0.17.17 in Python with default parameters. We projected the result to the t-distributed stochastic neighbor embedding (tSNE) plot from Seurat clustering analysis for visualization consistency. The Python package decoupler did pseudo-bulk functional analysis with default processing pipeline and parameters, in which we analyzed pathway-activity inferences based on PROGENy, a comprehensive resource containing a curated collection of pathways and their target genes with weights for each interaction.

### Serum stimulation

WBCs from HDs were resuspended in a mixture of 70% RPMI 1640 medium and 30% serum obtained from either HDs or patients with septic shock. Lipopolysaccharide (LPS) was added to achieve a final 10 ng/mL concentration. A total of 1 mL of the cell suspension was plated in each well of a 24-well culture plate. After a 24-hour stimulation period, we collected cells and centrifuged to form a pellet. Subsequently, the pellet was resuspended in an appropriate volume of TsingZol Reagent, ensuring thorough mixing to promote cell lysis and RNA stabilization. IL-1B and IL-6 gene expression levels were quantified using real-time RT-qPCR.

### Stimulation with recombinant human CD121b protein

WBCs from HDs were resuspended in complete RPMI 1640 medium supplemented with 10% fetal bovine serum (FBS). LPS was added to a final concentration of 10 ng/mL and recombinant human CD121b protein at a final concentration of 500 ng/mL. Following a 24-hour stimulation period, cells were collected as previously described. Real-time RT-qPCR assessed the expression levels of IL-1B and IL-6.

### Antibody blockade of CD121b

WBCs from HDs were resuspended in a mixture of 70% RPMI 1640 medium and 30% serum derived from patients with septic shock. LPS was added to achieve a final concentration of 10 ng/mL and an anti-human CD121b blockade antibody at 10 µg/mL. After a 24-hour stimulation period, cells were collected following previously described protocols. IL1B, IL6, IL8, and TNF expression levels were analyzed using real-time RT-qPCR.

### Drug treatments of cells

WBCs isolated from patients with septic shock were resuspended in complete RPMI 1640 medium supplemented with 10% FBS. Several pharmacological agents were selected for cell stimulation, including L-arginine (final concentration: 1 mM), dexamethasone (final concentration: 100 μM), and ATRA (final concentration: 50 μM). Stimulation experiments were conducted with and without adding human granulocyte-colony stimulating factor (G-CSF) at a final concentration of 50 ng/mL. Following a 24-hour stimulation period, cells were collected according to the aforementioned procedures for subsequent single-cell RNA sequencing. The gene expression levels of MME and IL-1R2 were assessed by real-time RT-qPCR.

### Statistical analyses

Statistical analyses were performed using GraphPad Prism 8.0 (GraphPad, La Jolla, CA, USA). Data normality was assessed with the Shapiro–Wilk test, while homogeneity of variance was evaluated using the Brown–Forsythe test. *For normally distributed and homoscedastic data*, statistical comparisons were made using a 2-tailed Student’s t-test, paired t-test, or one-way ANOVA, followed by Bonferroni *post hoc* correction. Data are expressed as mean ± standard error of the mean (SEM). In cases where the data did not meet normality assumptions, results are presented as median with interquartile range (IQR) and analyzed using the Mann–Whitney U test or the Kruskal–Wallis test, followed by Dunn’s *post hoc* test. A P value of less than 0.05 was considered statistically significant.

### Role of funders

The funding sources did not influence the study’s design, data collection, data analysis, interpretation of results, or manuscript writing.

## Result

### CD66b^+^CD10^-^ neutrophils are associated with the progression of septic shock

To investigate the impact of the immune response on the progression of sepsis, we retrospectively assessed the proportions and absolute numbers of various immune cell subsets in the peripheral blood of HDs and patients in the ICU. A significant increase in the proportion of neutrophils was observed in septic shock patients, accompanied by a corresponding decrease in lymphocytes and monocytes proportions (**Supplementary Figure 1A**). Consistent with previous studies (15), the neutrophil count in patients with septic shock was significantly elevated (**Supplementary Figure 1B**). These results implied that neutrophils may play a key role in the pathogenesis of septic shock.

**Figure 1:**
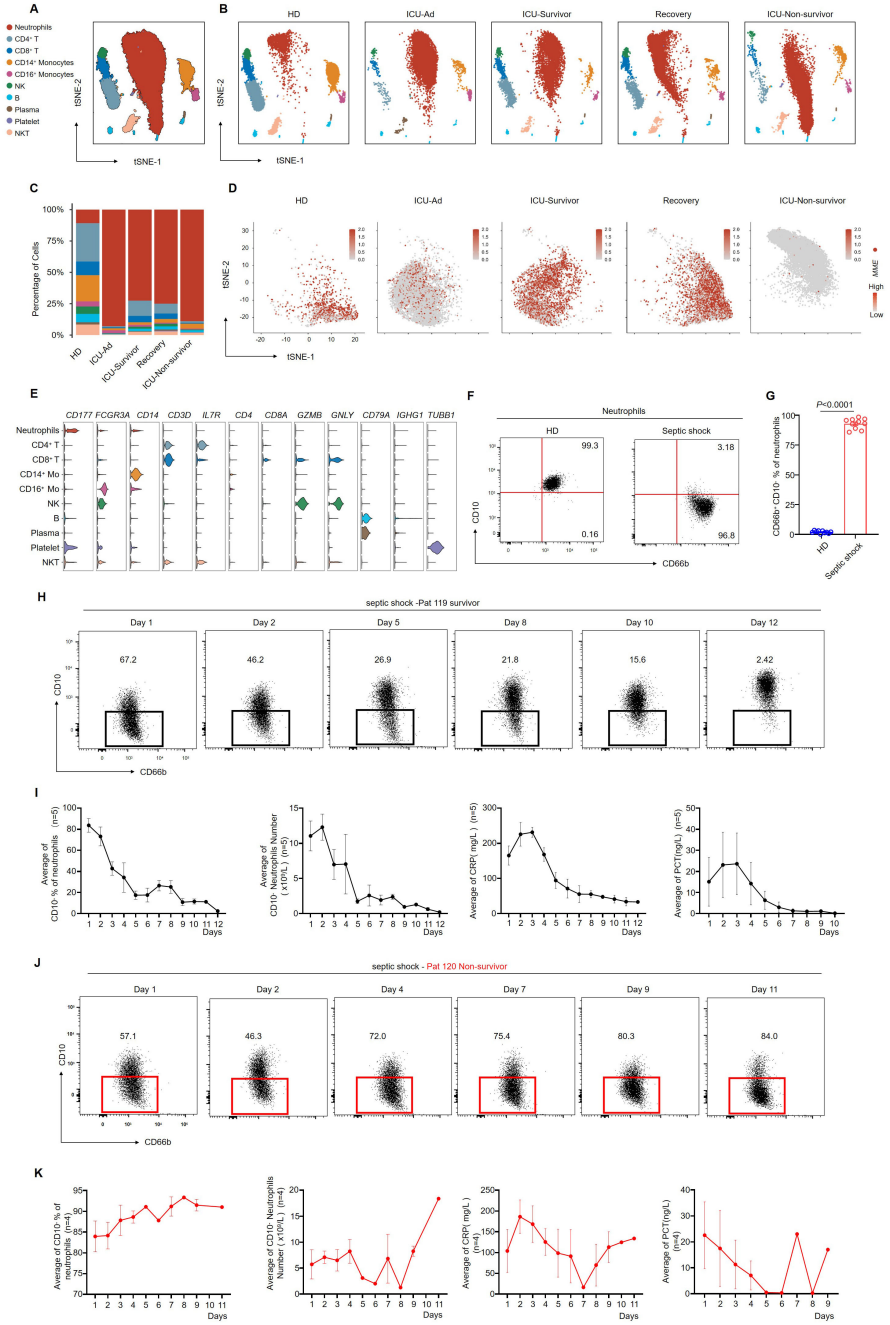
CD66b^+^ CD10^−^ neutrophils are related to the progression of septic shock. **(A)** the t-SNE plot of single-cell sequencing data (33,472 cells) from peripheral blood of patients with septic shock and HDs, annotated by distinct cell populations. **(B)** t-SNE plots for individual sample groups. HDs, n = 3; ICU Admission (ICU-Ad) & ICU-Survivor, n = 1; Recovery, n = 1; ICU Non-survivor, n = 1. **(C)** Changes in the proportions of immune cell subpopulations across conditions. **(D)** *MME* expression pattern projected on the t-SNE plot, showing neutrophils only. **(E)** Violin plots displaying a scaled expression of selected signature genes across clusters. **(F)** Representative flow cytometry plots showing the frequencies of CD66b^+^CD10^−^ neutrophils in healthy donors (left) and a patient with septic shock (right). **(G)** Quantification of CD66b^+^CD10^−^ neutrophils in healthy donors (n = 10) and septic shock patients (n = 10). **(H)** Dot plots showing dynamic changes in the proportion of CD66b^+^CD10^-^ neutrophils in surviving septic shock patients. **(I)** Dynamic changes in the average proportion of CD10^−^ neutrophils, absolute counts of CD10^−^ neutrophils, serum CRP, and PCT levels in septic shock survivors (n = 5). **(J)** Dot plots showing dynamic changes in the proportion of CD66b^+^CD10^-^ neutrophils in septic shock patients who died. **(K)** Dynamic changes in the average proportion of CD10^-^ neutrophils among total neutrophils, absolute CD10^−^ neutrophil counts, serum CRP, and PCT levels in patients who succumbed to septic shock (n = 4). Three or more independent experiments were performed. Statistical analyses were performed using two-tailed unpaired Student’s t-test **(G)**.

To further investigate the characteristics of peripheral blood immune cells in septic shock patients, we collected blood samples from various groups for comparative analysis. The study cohort consisted of three HDs, one patient shortly after ICU admission (ICU-Ad) and the same patient upon clinical improvement at ICU discharge (ICU-Survivor), one septic shock patient discharged from the hospital (Recovery), and one septic shock patient who deteriorated and subsequently passed away shortly after ICU admission (ICU-Non-survivor). Single-cell RNA-seq analysis revealed a significant increase in neutrophil counts and a notable reduction in other immune cell populations in ICU patients compared to the HDs. This trend was even more pronounced in the non-survivor group (**Figures 1A–C, E**). We clustered single-cell sequencing data. We focused on neutrophil subsets from different samples for dimensionality reduction analysis. We observed increased expression of *MME*, which encodes CD10, in neutrophils from HDs.

*In contrast*, neutrophils from ICU-Ad and ICU-Non-survivor exhibited reduced *MME* expression. At the same time, a significant increase was observed in ICU-Survivor and the Recovery group (**Figure 1D**). Based on these observations; we conducted bulk sequencing of neutrophils isolated from three HDs and four septic shock patients at various stages of illness: upon ICU admission (ICU-Ad), during ICU hospitalization (ICU-Hosp), and before ICU discharge following clinical improvement (ICU-Survivor). Principal component analysis (PCA) revealed significant transcriptomic differences between the HDs, ICU-Ad, ICU-Hosp, and ICU-Survivor **Supplementary Figure 2A**).

Flow cytometry analysis of CD10 expression on neutrophils revealed elevated CD10 levels in HDs. At the same time, septic shock patients exhibited markedly reduced CD10 expression (**Figures 1F, G**; **Supplementary Figure 3A**). To further investigate the association between CD10 expression and disease severity, we performed longitudinal monitoring of neutrophil phenotypes in two septic shock patients. Patient 119 showed clinical improvement, with a corresponding decrease in the proportion of CD66b^+^CD10^-^ neutrophils, ultimately leading to discharge from the ICU (**Figure 1H**). In surviving patients, a decrease in CD10^−^ neutrophils was associated with recovery and a reduction in inflammatory markers, including C-reactive protein (CRP) and procalcitonin (PCT) (**Figure 1I**). In contrast, Pat 120 exhibited a sustained increase in the proportion of CD66b^+^CD10^-^ neutrophils, which remained elevated as the patient’s condition progressively deteriorated (**Figure 1J**). Analysis of clinical data from deceased septic shock patients revealed persistently elevated levels of CD10^−^ neutrophils, which were associated with increased inflammatory markers, worsening disease progression, and eventual death (**Figure 1K**). These results suggested a strong association between CD66b^+^CD10^−^ neutrophils and the severity of septic shock.

### Arrest of neutrophilic development in patients with septic shock

**Figure 2:**
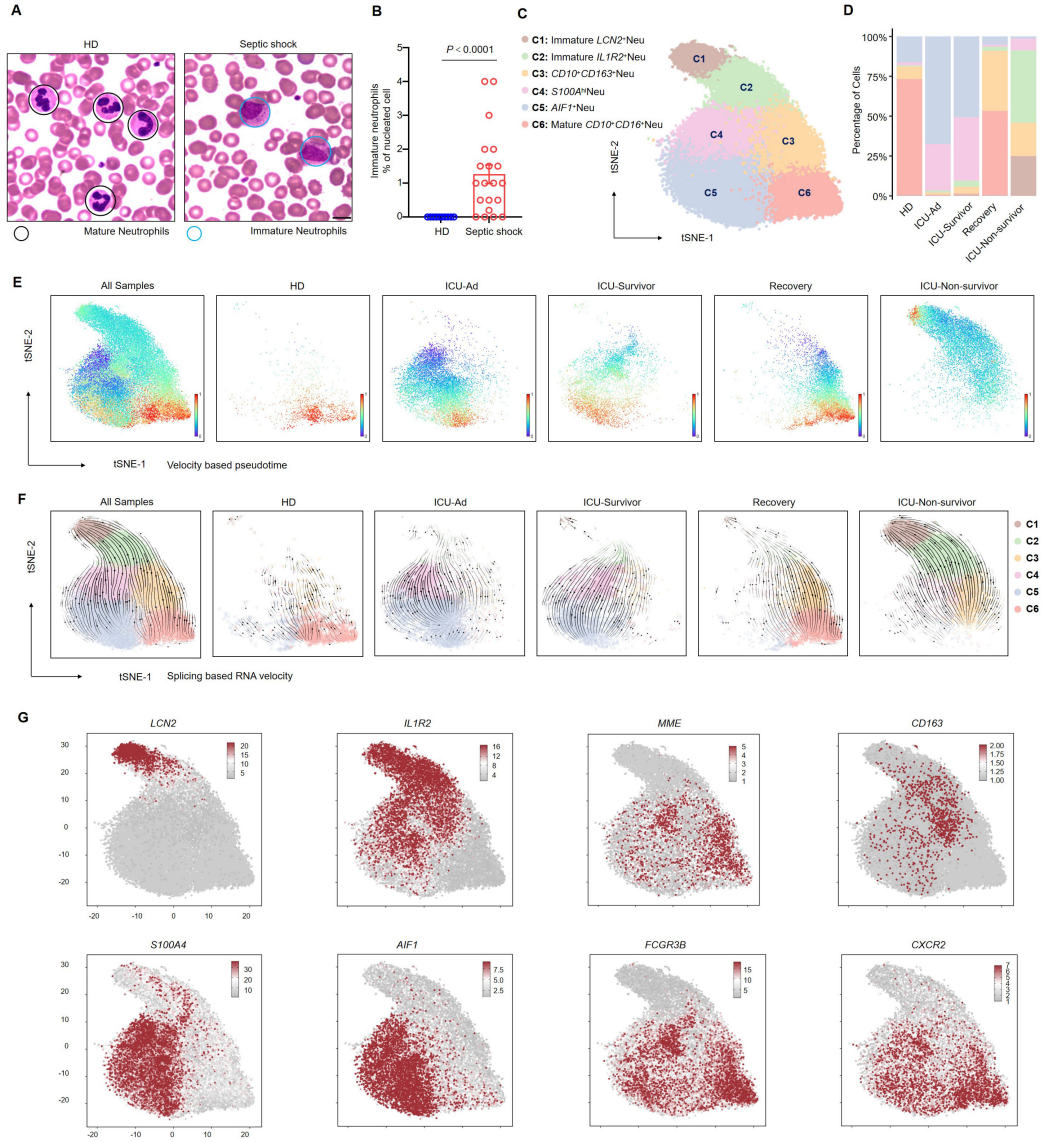
Blockade of the development of CD66b^+^CD10^+^ neutrophils in septic shock. **(A)** Representative Wright–Giemsa–stained peripheral blood smears showing mature neutrophils in a healthy donor (left) and immature neutrophils in a patient with septic shock (right); scale bar, 10 μm. **(B)** The ratio of the proportion of immature neutrophils to nucleated cells in the peripheral blood of healthy donors (n = 10) and septic shock patients (n = 20). **(C)** t-SNE plot identifying six neutrophil clusters. **(D)** Proportions of neutrophil clusters across five patient groups. **(E, F)** Latent time analysis **(E)** and neutrophil RNA velocity **(F)** are displayed for each sample group on the t-SNE plot. **(G)** t-SNE plots illustrating the expression of selected marker genes across six neutrophil clusters. Statistical analyses were performed using the Mann–Whitney test **(B)**.

We performed Wright-Giemsa staining on peripheral blood smears to investigate the morphological characteristics of neutrophils in patients with septic shock. The results show a significant increase in immature neutrophils, including myelocytes and metamyelocytes, in patients with septic shock (**Figures 2A, B**). To further investigate neutrophil subpopulations in the peripheral blood of septic shock patients, neutrophils were isolated from all samples for comprehensive follow-up analyses. Cell clustering analysis identified six distinct neutrophil clusters (**Figure 2C**). The distribution of these clusters is illustrated in a percentage bar chart (**Figure 2D**). The neutrotime signature (16) reveals a progressive maturation of neutrophils from clusters C1 to C6 (**Supplementary Figure 4A**). Notably, significant enrichment of immature neutrophils was observed in ICU-Ad and the ICU-Non-survivor groups compared to HDs and Recovery group. Conversely, as patient conditions improved (from ICU-Survivor to Recovery), the proportion of C6 increased (**Figure 2E**). Neutrophil development was blocked in ICU-Ad and ICU-Non-survivor patients. RNA velocity analysis further elucidated a distinct developmental trajectory from C1 to C6 (**Figure 2F**). We identified well-established marker genes for neutrophil maturation (MME, CXCR2, FCGR3B) within sequencing data, revealing that the C6 clusters exhibited the highest degree of maturation. C1 and C2 clusters exhibited elevated expression of LCN2 (17) and IL1R2 (13), while C3, C4, and C5 clusters, which represent an intermediate evolutionary state, demonstrated characteristic expression of CD163 (18), S100A4 (19), and AIF1 (20) (**Figure 2G**). These results indicate that the blockade of the maturation and differentiation pathways in circulating neutrophils was an essential feature of the immune response in patients with septic shock.

### Identification of CD121b^+^ neutrophils in septic shock

**Figure 3:**
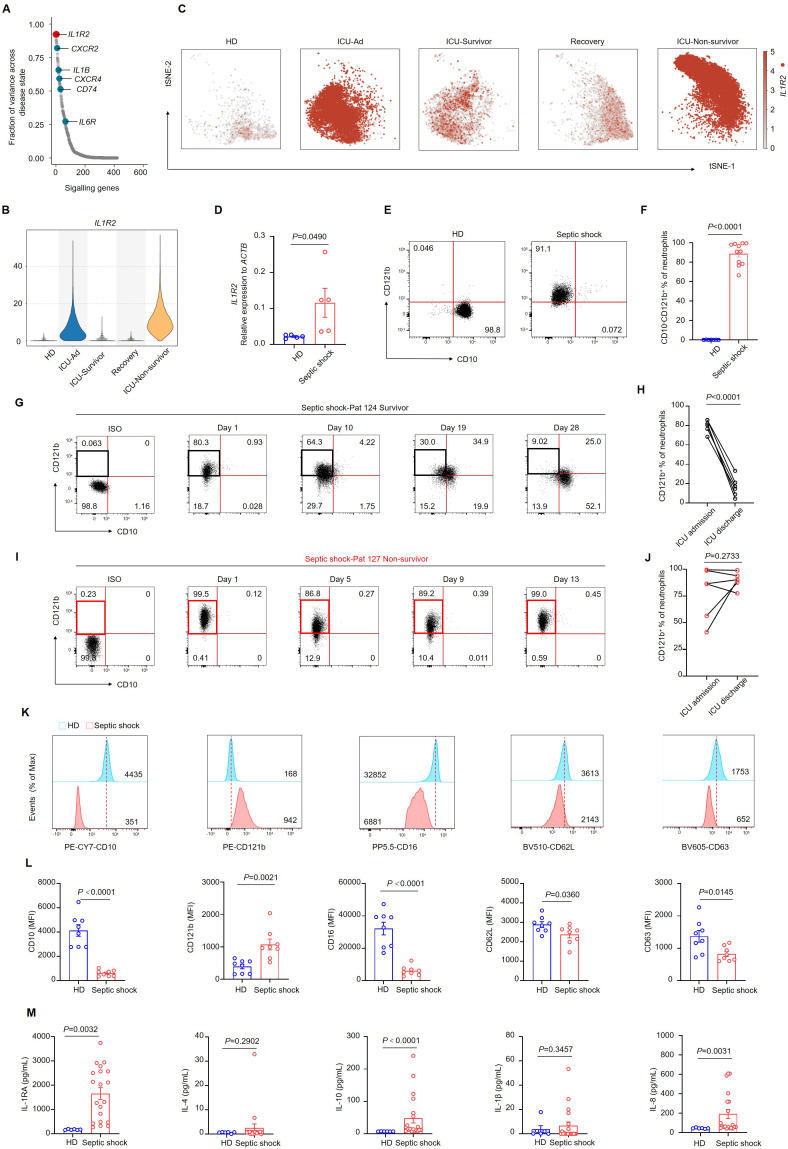
CD10^−^ CD121b^+^ neutrophils are positively correlated with mortality risk in septic shock. **(A)** Differential gene expression in neutrophils across distinct phases of sepsis, organized by the magnitude of change, with *IL1R2* showing the most pronounced alteration. **(B)** Violin plot depicting *IL1R2* expression in neutrophils across different phases of sepsis and healthy donors. **(C)** *IL1R2* expression projected on the t-SNE plot, highlighting neutrophils. **(D)** *IL1R2* expression in peripheral blood neutrophils from HDs (n = 5) and patients with septic shock (n = 5). **(E)** Representative flow cytometry plots showing CD10^−^CD121b^+^neutrophil frequencies in a healthy donor (left) and a patient with septic shock (right). **(F)** Quantification of CD10^−^ CD121b^+^ neutrophils in healthy donors (n = 7) and septic shock patients (n = 11). **(G, H)** Representative flow cytometry plots **(G)** and statistical analysis **(H)** showing the dynamics of CD10^−^ CD121b^+^ neutrophil frequencies in septic shock survivors from ICU admission to discharge (n = 6). **(I, J)** Representative flow cytometry plots **(I)** and statistical analysis **(J)** of CD10^−^ CD121b^+^ neutrophils in patients with septic shock leading to death (n = 6). **(K)** Histograms of CD10, CD121b, CD16, CD62L, and CD63 expression in peripheral blood neutrophils from healthy donors (upper) and septic shock patients (lower). **(L)** Mean fluorescence intensity (MFI) of CD10, CD121b, CD16, CD62L, and CD63 in neutrophils from healthy donors (n = 8) and septic shock patients (n = 8). **(M)** Serum concentrations of IL-RA, IL-4, IL-10, IL-1β, and IL-8 in healthy donors (n = 6) and septic shock patients (n = 20). Three or more independent experiments were conducted. Statistical analyses were performed using two-tailed unpaired Student’s t-test **(D, F, K)**, paired t-test **(H, J)**, and Mann–Whitney test **(L)**.

We aimed to investigate the genes implicated in the progression of sepsis within neutrophils. By integrating scRNA-sequencing data across various disease states, we assessed the correlations between different genes and disease progression. *IL1R2*, which encodes CD121b, was identified as one of the genes most closely correlated with sepsis progression (**Figures 3A–C**). Furthermore, we demonstrated a significant upregulation of *IL1R2* expression in patients with septic shock at mRNA and protein levels (**Figures 3D–F**). Consistent with our previous findings, bulk RNA sequencing data revealed distinct transcriptional profiles among the HDs, ICU-Ad, ICU-Hosp, and ICU-Survivor (**Supplementary Figure 5A**). Additionally, we documented alterations in the expression of genes from the *S100A*, *MMP* and *IL1* families across these groups (**Supplementary Figures 5B–D**). Notably, the changes in *IL1R2* expression paralleled those observed at the single-cell level, further highlighting the characteristic upregulation of *IL1R2* in patients with septic shock (**Supplementary Figure 5D**). These findings indicated that genes from these families were expressed at elevated levels in ICU-Ad and ICU-Hosp, returning to baseline levels in ICU survivors.

Furthermore, we utilized flow cytometry to dynamically analyze neutrophil phenotypes in patients who improved to those who deteriorated and subsequently died. The proportion of CD10^-^ CD121b^+^ neutrophils decreased progressively in septic shock patients who exhibited clinical improvement from ICU admission to discharge (**Figures 3G, H**). In contrast, patients who deteriorated and ultimately died exhibited a sustained elevation of CD10^-^ CD121b^+^ neutrophils throughout their hospitalization, with levels remaining high until death (**Figures 3I, J**). These data suggested a correlation between the reduction of CD10^−^CD121b^+^neutrophils and clinical improvement in septic shock patients. To evaluate the chemotactic, activation, and degranulation functions of neutrophils in patients with septic shock, we utilized flow cytometry to analyze the expression of CD10, CD121b, CD16, CD62L, and CD63 on neutrophils from HDs and septic shock patients. Our findings demonstrated that septic shock patients exhibited a significant reduction in CD10 and CD16 expression compared to HDs, while CD121b expression was notably elevated. These findings suggest impaired neutrophil maturation in septic shock patients. Additionally, the observed downregulation of CD62L and CD63 indicates compromised migratory and degranulation functions of neutrophils (**Figures 3K, L**). In contrast, serum levels of IL-10, IL-1RA, and inflammatory cytokines (including IL-8) were significantly elevated in septic shock patients compared to HDs (**Figure 3M**).

### CD121b^+^ neutrophils exhibit immunosuppressive features

**Figure 4:**
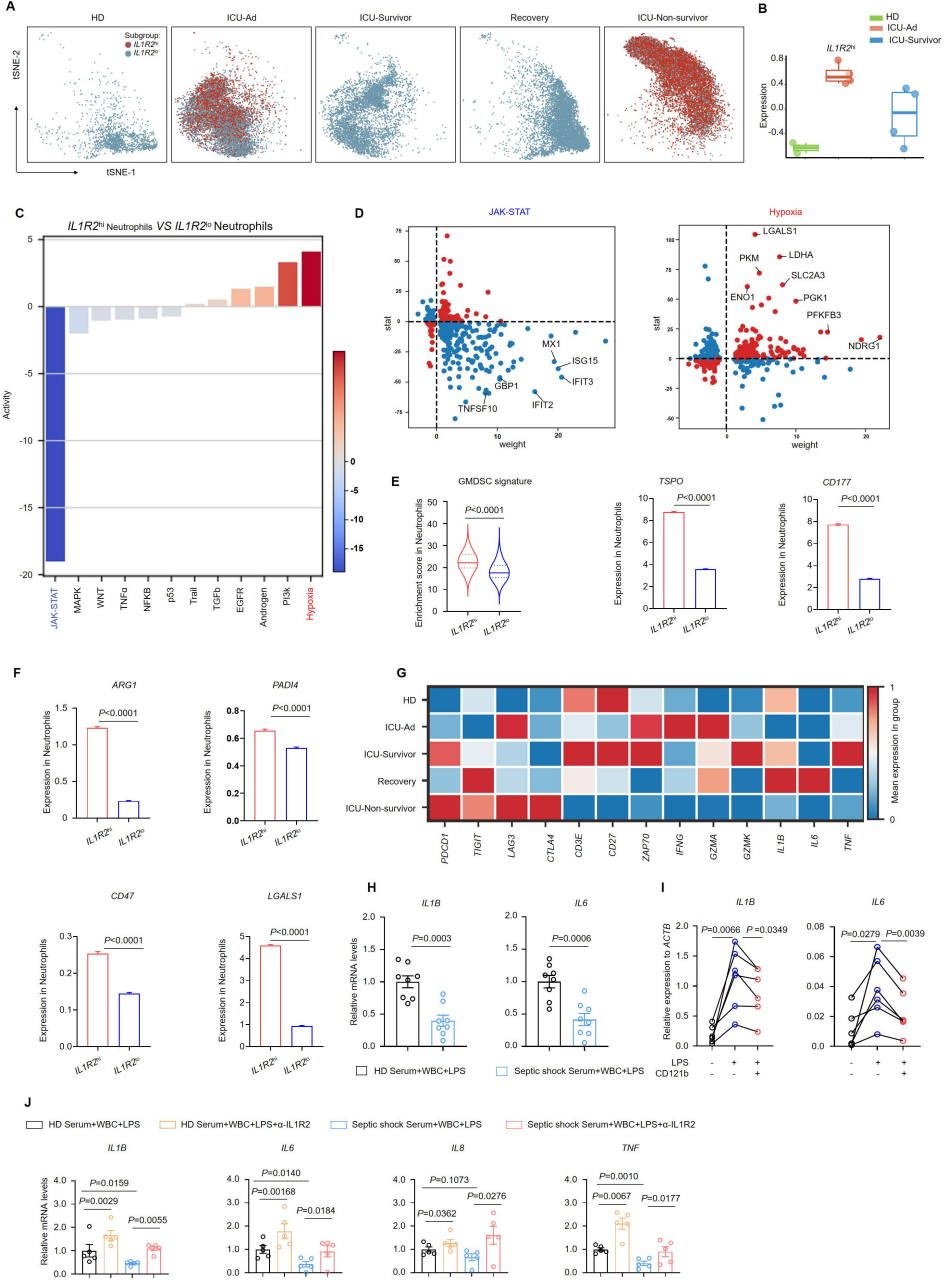
CD121b^hi^ neutrophils have an immunosuppression role in septic shock. **(A)** t-SNE plot displaying two distinct neutrophil populations separated by *IL1R2* expression across five sample groups. **(B)** Based on RNA-sequencing data, a bar graph illustrating gene-signature enrichment scores of highly expressed IL1R2 in neutrophils. **(C)** Inferred pathway activity among neutrophil subclusters using the PROGENy platform. **(D)** Differentially expressed genes specific to the JAK-STAT and hypoxia signaling pathways. **(E)** Enrichment scores of the GMDSC signature in neutrophil subclusters, with bar graphs showing variation in the expression of *TSPO* and *CD177*. **(F)** Bar graphs depicting variation in the expression of immunosuppressive genes in neutrophils. **(G)** Heatmap displaying a differential expression of immune-related genes in T cells across five sample groups. **(H)** Gene expression of *IL1B* and *IL6* in white blood cells from HDs following 24 h of LPS stimulation in serum from HDs (n = 8) or septic shock patients (n = 8). **(I)** Gene expression of *IL1B* and *IL6* in white blood cells from HDs (n = 6) following 24 h of LPS stimulation in FBS with or without recombinant human CD121b protein. **(J)** Gene expression of *IL1B*, *IL6*, *IL8*, and *TNF* in white blood cells from HDs following 24 h of LPS stimulation in serum from HDs (n = 5) or septic shock patients (n = 5) with or without the addition of CD121b (IL-1R2)-blocking antibodies. Three or more independent experiments were performed. Statistical analyses were conducted using two-tailed unpaired Student’s t-test **(E, F, H)**, paired t-test **(I, J)**, and Mann-Whitney test **(J)**.

We defined cells with counts exceeding the upper quartile as “*IL1R2* ^hi^netrophils” in our single-cell analysis. Among the five groups, neutrophils from HDs, ICU-Survivor, and Recovery patients predominantly exhibited the *IL1R2* ^lo^ phenotype. In contrast, neutrophils from ICU-Ad and ICU-Non-survivor primarily exhibited the *IL1R2* ^hi^ phenotype (**Figure 4A**). Utilizing the characteristic genes of *IL1R2* ^hi^ neutrophils as a gene set, we performed gene set variation analysis to assess the enrichment of this gene set in bulk sequencing data from neutrophils of HDs, ICU-Ad, and ICU-Survivor. We observed a significantly higher enrichment score for this gene set in the ICU-Ad (**Figure 4B**). These findings indicate a strong association between *IL1R2* ^hi^ neutrophils and the disease state, suggesting that this subpopulation may serve as a predictive biomarker for disease progression. Further analysis of the *IL1R2* ^hi^ and *IL1R2* ^lo^ neutrophils subsets revealed differentially expressed genes, and pathway-activity inferences were derived from the PROGENy resource, identifying distinct signaling pathways (**Figures 4C, D**). In the *IL1R2* ^hi^ subgroup, we observed upregulated of signaling pathways such as “TGF-β”, “Androgen”, “PI3K”, and “Hypoxia”, while pathways including “JAK-STAT”, “NF-κB”, “TNF-α”, and “P53” was downregulated. The expression of downstream genes associated with the JAK-STAT pathway, including *IFIT2*, *GBP1*, *ISG15*, and *IFIT3*, was significantly decreased. These findings suggest a disruption of the interferon signaling in the *IL1R2* ^hi^ subgroup.

Granulocytic myeloid-derived suppressor cells (G-MDSCs) are recognized as a subset of neutrophils characterized by their significant immunosuppressive properties, albeit with distinct features (21, 22). To evaluate the immunophenotype of neutrophils in sepsis, we utilized a gene set representative of G-MDSCs (23). Upon scoring the two previously mentioned subsets, we found that *IL1R2* ^hi^ neutrophils exhibited more pronounced GMDSC-like characteristics, evidenced by significantly upregulated expression of the signature genes *TSPO* and *CD177* (**Figure 4E**). To investigate the functional characteristics of *IL1R2* ^hi^ neutrophils, we assessed the expression of classical immunosuppressive molecules in both *IL1R2* ^hi^ neutrophils and *IL1R2* ^lo^ neutrophils using single-cell sequencing data. Notably, we observed significant upregulation of genes associated with immunosuppressive functions, including *ARG1*, *PADI4*, *CD47*, and *LGALS1*, in the *IL1R2* ^hi^ subgroup (**Figure 4F**), indicating enhanced expression of immunosuppressive genes in these neutrophils. Furthermore, we analyzed the expression of key immune checkpoint molecules on T cells and markers associated with T-cell activation. We found that major immune checkpoint molecules, such as *PDCD1*, *TIGIT*, and *LAG3*, were upregulated in T cells from deceased patients, while expression of activation markers, including *CD3E*, *ZAP70*, and *CD27*, was downregulated. The expression of functional markers such as IFNG, GZMA, and GZMK, along with the capacity to produce cytokines including IL1B, IL6, and TNF, was significantly downregulated (**Figure 4G**). These molecules are critical in mediating T-cell activation pathways in response to antigenic stimulation (24). The observed reduction in intercellular communication may reflect compromised coordination among immune cells and a diminished efficacy of the immune response (25). We isolated peripheral blood white blood cells (WBCs) from HDs and stimulated them with LPS *in vitro*. In the experimental group, we supplemented the serum from septic shock patients and co-cultured for 24 hours. Subsequent RT-PCR analysis of the WBCs revealed a marked suppression of proinflammatory gene expression, including IL1B and IL6, in cells exposed to serum from critically ill patients (**Figure 4H**). The results indicate the presence of immunosuppressive factors in the serum of septic shock patients. To further explore whether CD121b could inhibit the transduction of inflammatory signals and exert immunosuppressive effects, we isolated peripheral blood immune cells from HDs and stimulated them with LPS *in vitro*. In this experimental setup, soluble CD121b (sCD121b) protein was added, and the cells were co-cultured for 24 hours. RT-qPCR analysis demonstrated significant downregulation of *IL1B* and *IL6* expression in the experimental group (**Figure 4I**). Following the introduction of a blocking antibody against CD121b, we observed a reversal of the serum immunosuppressive effects, with restored expression of *ILB*, *IL6*, *IL8*, and *TNF*. These findings strongly suggested that CD121b was a major mediator of immunosuppressive responses in septic shock (**Figure 4J**). Our data confirmed that the CD121b protein was a key source of immunosuppressive signals in patients with septic shock, indicating that *IL1R2* ^hi^ neutrophils may have contributed to this immunosuppression in severe sepsis.

### ATRA rescues the immunosuppressive effect of neutrophils

**Figure 5:**
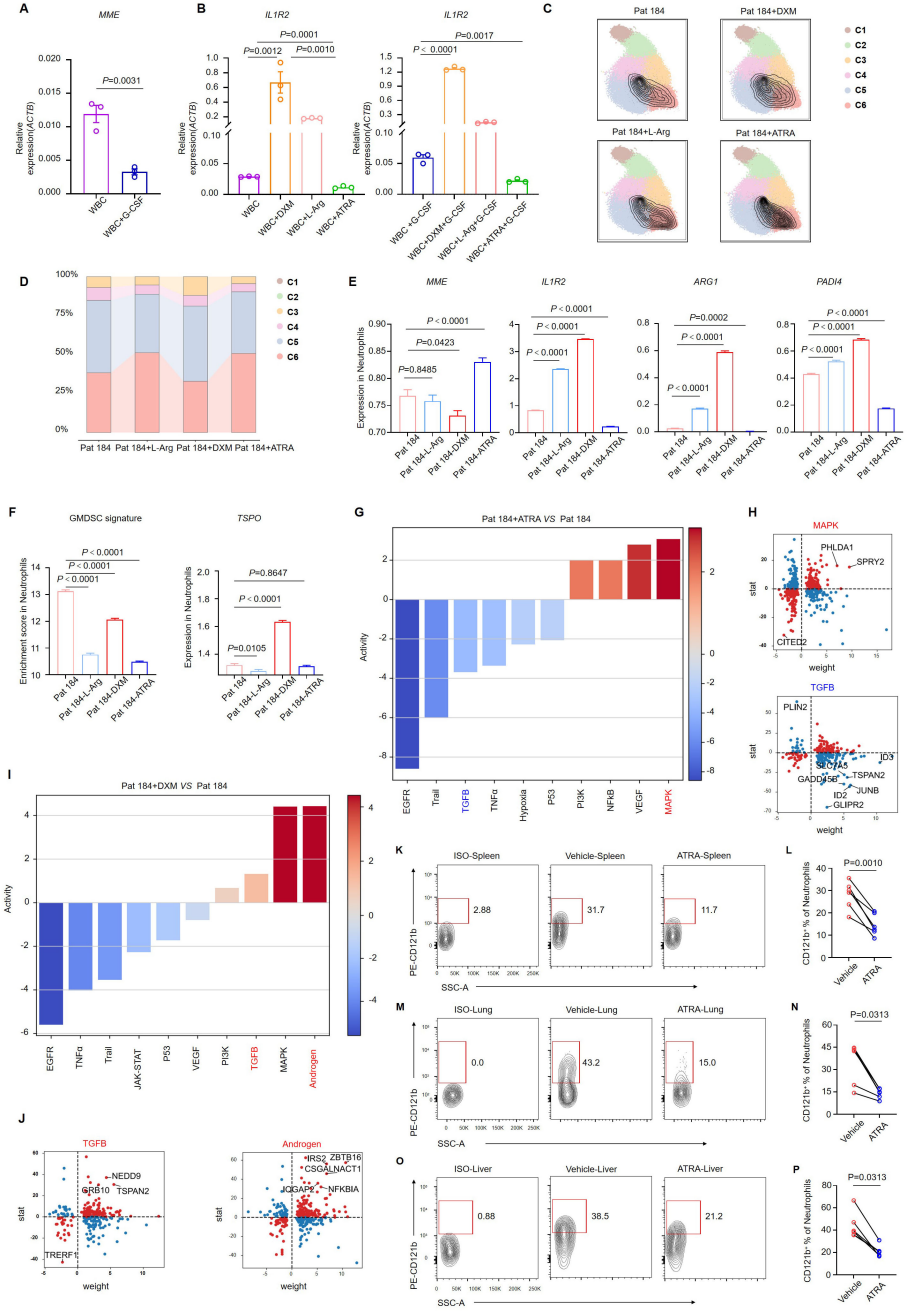
ATRA alleviates the immunosuppressive effect of neutrophils. **(A)** *MME* expression in white blood cells from septic shock patients (n = 3) after 24 h of G-CSF stimulation. **(B)** *IL1R2* expression in white blood cells from septic shock patients (n = 3) following 24 h of stimulation with dexamethasone, L-arginine, or ATRA, with or without G-CSF. **(C)** Single-cell sequencing of neutrophils from septic shock patients after *in vitro* stimulation with DXM, L-Arg, or ATRA, with neutrophil subpopulations displaying maturation features circled in black. **(D)** Changes in the proportions of neutrophil subclusters. **(E)** Bar graphs showing variation in the expression of key genes in neutrophils of single-cell sequencing data. **(F)** GMDSC signature enrichment in neutrophils, with a bar graph showing variation in *TSPO* expression of single-cell sequencing data. **(G, H)** Pathway activity changes **(G)** and differentially expressed genes **(H)** in the MAPK and TGF-β signaling pathways in neutrophils treated with ATRA. **(I, J)** Pathway activity changes **(I)** and differentially expressed genes **(J)** in the TGF-β and androgen signaling pathways in neutrophils treated with DXM. **(K-O)** Representative flow cytometry plots showing the proportion of CD121b^+^ human neutrophils in the spleen **(K)**, lung **(M)**, and liver **(O)** of NCG mice 24 h after ATRA administration. **(L, N, P)** Statistical analysis of CD121b^+^ human neutrophil proportions in the spleen **(L)**, lung **(N)**, and liver **(P)** of vehicle-treated (n = 6) and ATRA-treated (n = 6) NCG mice. Three or more independent experiments were performed. Statistical analyses were conducted using two-tailed unpaired Student’s t-test **(A, E, F)**, one-way ANOVA **(B)**, paired t-test **(L)**, and Wilcoxon test **(N, P)**.

ATRA, a naturally occurring vitamin A derivative, was crucial in regulating cell differentiation, proliferation, and apoptosis (26–28). To investigate whether ATRA could ameliorate the immunosuppressive state of patients by promoting neutrophil differentiation, we isolated WBCs from the peripheral blood of patients with septic shock and cultured them for 24 hours. In this culture system, we introduced ATRA, L-Arginine, and Dexamethasone. Additionally, we established a parallel system incorporating the same drug combinations, supplemented with G-CSF. We observed a reduction in the expression of *MME* in immune cells treated with G-CSF, indicating an enhanced generation of immature neutrophils (**Figure 5A**). Among the three drugs, both dexamethasone and L-Arginine upregulated the expression of *IL1R2* in neutrophils. Intriguingly, ATRA downregulated *IL1R2* expression regardless of adding G-CSF, suggesting its potential as a therapeutic agent for septic shock. (**Figure 5B**). we collected WBCs from four groups (Control, Dexamethasone, L-arginine, and ATRA) and performed single-cell sequencing to investigate this phenomenon further. We extracted neutrophil subpopulations from the sequencing data and mapped them onto single-cell sequencing data derived from neutrophils in human peripheral blood (**Figures 2C**, **5C**). Contour plots revealed an increase in the proportion of C6 neutrophils following ATRA stimulation, indicating that ATRA facilitated the development and maturation of neutrophils of septic shock patients. In contrast, the proportion of C6 neutrophils decreased after dexamethasone stimulation, suggesting that dexamethasone inhibited neutrophil maturation (**Figure 5C**). Based on this subgrouping result, we generated a bar graph depicting the percentages of C1–C6 subsets across the four sample groups (**Figure 5D**).

We extracted neutrophil datasets from the single-cell sequencing data of the four samples and labeled the expression of four key molecules: *MME*, *IL1R2*, *ARG1*, and *PADI4*. We observed upregulation of *MME* expression in the ATRA group, indicating that ATRA induced neutrophil maturation. Additionally, IL1R2, ARG1, and PADI4 expression were downregulated following ATRA stimulation, suggesting that ATRA reversed the immunosuppressive characteristics of neutrophils in patients with septic shock. *IL1R2* expression was upregulated after dexamethasone stimulation, which aligned with our understanding of the immunosuppressive function of dexamethasone (**Figure 5E**). These results demonstrated the potent *in vitro* effects of ATRA in restoring neutrophil maturation and reversing immunosuppression. We utilized a characteristic gene set of GMDSC and the feature gene *TSPO* to assess the immunosuppressive level of neutrophils (**Figure 5F**). The immunosuppressive state of septic shock patients was reversed following ATRA induction. Moreover, we analyzed signaling-pathway enrichment based on differentially expressed genes in neutrophils. After ATRA stimulation, the expression of the inflammatory signaling pathways, including MAPK and NF-κB, was enhanced, indicating neutrophil activation. Concurrently, the expression of the TGF-signaling and hypoxia-signaling pathways was downregulated, suggesting a weakened immunosuppressive effect and the restoration of neutrophil metabolic capacity alongside impaired function (**Figure 5G**). Detailed analysis of the differentially expressed genes within these signaling pathways consistently revealed similar phenomena (**Figure 5H**). Next, we examined the gene expression changes following dexamethasone stimulation. In contrast to ATRA, dexamethasone stimulation markedly increased immunosuppressive signals, notably the TGF-β and androgen signaling pathways, both exhibited significant upregulation (**Figures 5I, J**).

We established a sepsis-humanized mouse model to investigate whether ATRA could reduce CD121b expression on neutrophils. Immune cells from septic shock patients were transferred to severely immunodeficient mice (NCG), which were subsequently treated with ATRA. Twenty-four hours post-ATRA administration, we collected the mice’s spleen, lung, and liver for flow cytometry analysis. Compared to the vehicle group, CD121b expression in human neutrophils was downregulated in the ATRA-treated group (**Figures 5K-P**). These results indicated that ATRA enhanced the function and development of neutrophils *in vitro* and *in vivo*.

### CD121b can be a potential indicator of immunosuppression in septic shock

**Figure 6:**
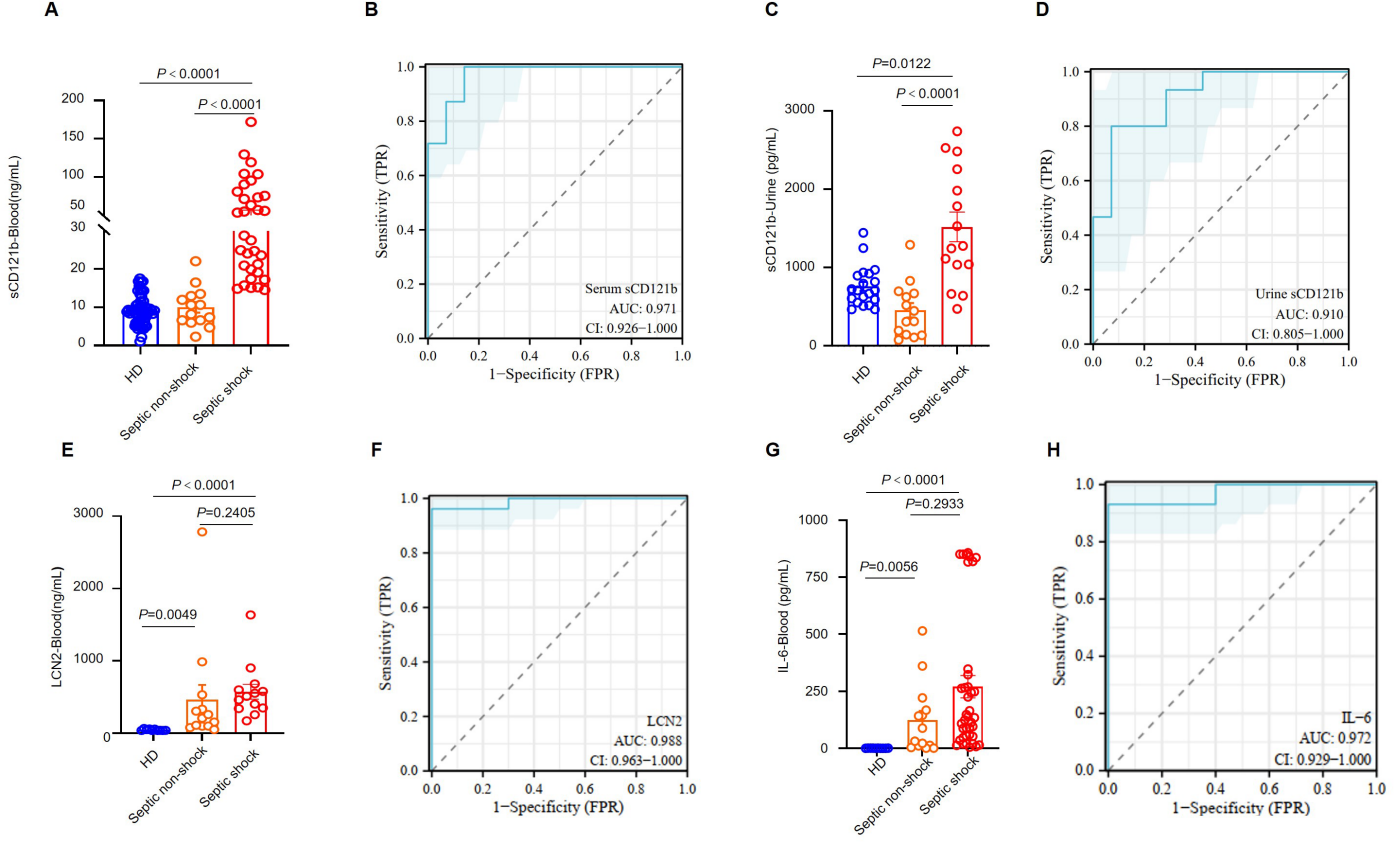
CD121b as a biomarker for the immunosuppressive state in patients with septic shock. **(A)** Serum levels of sCD121b in HDs (n = 57), non-septic patients (n = 14), and septic shock patients (n = 39). **(B)** The ROC curve for serum CD121b levels comparing non-septic patients and septic shock patients. **(C)** Urine levels of sCD121b in HDs (n = 20), non-septic patients (n = 14), and septic shock patients (n = 15). **(D)** ROC curve for urine CD121b levels comparing non-septic and septic shock patients. **(E)** Serum levels of LCN2 in HDs (n = 10), non-septic patients (n = 13), and septic shock patients (n = 13). **(F)** ROC curve for serum LCN2 levels comparing HDs and sepsis patients (including non-septic and septic shock patients). **(G)** Serum levels of IL-6 in HDs (n = 10), non-septic patients (n = 14), and septic shock patients (n = 39). **(H)** ROC curve for serum IL-6 levels comparing HDs and sepsis patients (including non-septic and septic shock patients). Three or more independent experiments were performed. Statistical analyses were performed using the Kruskal-Wallis test **(A, C, E, G)** and AUC analysis **(B, D, F, H)**.

We evaluated CD121b expression on various immune cell types in peripheral blood. CD121b was minimally expressed in healthy donors’ and septic shock patients’ T cells. To determine whether neutrophils could produce sCD121b, we cultured neutrophils isolated from healthy donors and septic shock patients *in vitro*. Neutrophils derived from septic shock patients demonstrated the capacity to produce sCD121b (**Supplementary Figures 6A–C**). This observation supports that neutrophil are a source of immunosuppressive signaling in septic shock. To evaluate whether CD121b could function as a serum marker for immunosuppression, we collected samples from three groups: HDs, septic non-shock, and septic shock. We quantified the concentration of sCD121b in each group using ELISAs. Notably, the serum levels of sCD121b were significantly elevated in patients with septic shock compared to both HDs and septic non-shock patients (**Figure 6A**). Additionally, receiver operating characteristic (ROC) curves were constructed to assess the diagnostic performance of sCD121b for distinguishing between septic non-shock and septic shock. The area under the ROC curve (AUC) was calculated to be 0.971, indicating a high diagnostic accuracy (**Figure 6B**). Furthermore, we obtained consistent results in urine samples (**Figure 6C**). We observed an elevation in the concentration of sCD121b in the urine of patients with septic shock, with an area under the ROC curve (AUC) of 0.910 (**Figure 6D**). Soluble CD121b detection in plasma and urine, and its close association with clinical outcomes in septic patients, suggest its potential as a non-invasive marker for sepsis-related complications. We also measured the concentrations of IL-6 and LCN2, establishing serum markers of sepsis (29, 30). Our results revealed a significant increase in LCN2 levels in the serum of septic shock patients; however, no significant differences were found between those with and without septic shock. (**Figures 6E, F**). Conversely, IL-6 levels were markedly low in the serum of HDs but showed a substantial increase in both septic non-shock and septic shock patients (**Figure 6G**). ROC analysis demonstrated the diagnostic efficacy of IL-6 in differentiating HDs from patients with sepsis, yielding an AUC of 0.972 (**Figure 6H**). While IL-6 is a reliable marker of inflammation, it does not adequately differentiate between immunosuppressed states in septic shock patients.

## Discussion

Sepsis is characterized by disrupted homeostasis in two opposing directions: excessive inflammation and immune suppression (31). This complex immune response evolves over time, with many septic patients quickly developing severe immunosuppression, which is linked to poor outcomes (32). *(i)* This study identified CD121b^+^ neutrophils as key mediators of immunosuppression in septic shock, strongly correlating with worse outcomes. Blockading of downstream CD121b signaling, either with a CD121b antibody or ATRA, CD121b expression in neutrophils was successfully downregulated both *in vitro* and in a humanized mouse model.

*(ii)* We performed single-cell sequencing of whole blood and transcriptomic analysis to dynamically track changes in immune cell composition throughout different stages of disease progression. In contrast to studies utilizing randomly selected samples (33, 34), our investigation focused specifically on patients with septic shock, emphasizing continuous monitoring throughout the disease. We observed a significant increase in immature neutrophils in these patients and a reduction in lymphocyte counts—a hallmark of immunosuppressive. Furthermore, the subset of CD10^-^ CD121b^+^ neutrophils was strongly associated with disease severity, and elevated CD121b expression on neutrophils emerged as a potential predictor of poor outcomes. In a study by Julian et al., two distinct sepsis response signature groups (SRS1 and SRS2) were identified (34). *Consistent with our results*, their single-cell sequencing data on sepsis revealed enrichment of CD121b^+^ neutrophils in patients with higher immunosuppression scores (SRS1 type) (13). Our study highlights the significant enrichment of CD10^-^ CD121b^+^ neutrophils in patients with poor prognoses characterized by an exaggerated septic response. We further validated these findings at the protein level through comprehensive analysis.

*(iii)* Research indicates that sCD121b exerts a more potent immunosuppressive effect than its membrane-bound counterpart (35, 36). sCD121b binds directly to interleukin-1 (IL-1) molecules, effectively inhibiting the intracellular transmission of IL-1 signaling (37). Our study demonstrates that sCD121b significantly reduces the expression of proinflammatory cytokines, including IL-6 and IL-1β, in immune cells. Conversely, the administration of CD121b-blocking antibodies disrupted the immunosuppressive effects observed in the serum of patients, compromising the anti-inflammatory response. The biological role of sCD121b signifies its potential as a marker for the transition from proinflammatory activation to anti-inflammatory suppression throughout the disease course. This observation underscores the promise of CD121b as a therapeutic target for enhancing immunosuppression in patients with septic shock.

*(iv)* Research also indicates that sepsis is characterized by immune system dysregulation. However, the distinction between excessive immune activation and immunosuppression remains ambiguous, directly impacting clinical diagnostic and therapeutic approaches. Our findings demonstrate that serum levels of sCD121b can effectively differentiate healthy donors from patients without septic shock and those with septic shock. Developing a molecular detection system for CD121b in serum, along with a potential non-invasive urine-based assay, could facilitate the assessment of immune status in patients with sepsis.

Recent investigations are exploring the potential of immunostimulatory agents for managing sepsis (7, 38), with a primary focus on cytokines (39–41), immune checkpoint inhibitors (42) (43), and immunoglobulins (44). *However*, these treatment modalities, which predominantly target adaptive immunity, often overlook the pivotal role of neutrophils, which dominate the immune landscape in the peripheral blood during the late stages of sepsis. Therefore, a promising strategy may involve ‘retraining’ immature neutrophils. ATRA, known for its ability to induce granulocyte maturation in acute promyelocytic leukemia treatment, presents a potential therapeutic candidate (45–48). Our findings demonstrate that ATRA enhances neutrophil maturation in the peripheral blood of patients, as evidenced by a significant reduction in CD121b expression at both the gene and protein levels. *The use* of glucocorticoids *in the treatment* of septic shock remains controversial, particularly concerning dosing, timing of initiation, and treatment duration (49–51). Notably, our data indicate that dexamethasone tends to drive neutrophils towards a more immature and immunosuppressive phenotype, which may explain why a substantial proportion of patients do not experience clinical benefit from this therapeutic approach.

*A primary limitation of our investigation* is the absence of single-cell data regarding bone marrow hematopoiesis, which is essential for elucidating the developmental origin of CD121b^+^ neutrophils. Further experimental evidence is needed to delineate the immunosuppressive functions of CD121b^+^ neutrophils, particularly concerning their direct interactions with lymphocytes. Furthermore, animal models and clinical trials will be necessary to determine the potential clinical benefits of ATRA.

## Conclusions

This study demonstrated that CD121b^+^ neutrophils positively correlated with septic shock severity. These cells displayed immunosuppressive characteristics; after blocking CD121b, proinflammatory cytokines increased in peripheral immune cells. Treatment with ATRA was found to down-regulate the expression of CD121b. CD121b may serve as a viable therapeutic target for intervention. These findings provide novel insights and directions for developing pharmacological strategies to mitigate immunosuppression in sepsis.

## Data Availability

The datasets presented in this study can be found in online repositories. The names of the repository/repositories and accession number(s) can be found below: HRA007054, HRA007050, HRA007060 (GSA; https://ngdc.cncb.ac.cn/gsa-human) (52, 53).

## Supplementary material

Supplementary Figure 1 (related to **Figure 1**). Increased proportion and absolute number of neutrophils in patients with septic shock. **(A)** Proportion neutrophils, lymphocytes, and monocytes in peripheral blood of HDs, patients surviving from septic shock (ICU-Survivor-Septic shock) and patients with septic shock leading to fatal outcome (ICU-Non-Survivor-Septic shock). n = 10, 8 and 10 for HD, ICU-Survivor-sepsis and ICU-Non-survivor-sepsis, respectively. **(B)** Cell count of neutrophils, lymphocytes, and monocytes in peripheral blood of HDs, non-sepsis ICU patients (ICU-non-Sepsis), patients without septic shock(ICU-Septic non-shock) and patients with septic shock (and ICU-Septic shock). n = 7, 37, 32 and 18 for HD, ICU-non-sepsis, ICU-Septic non-shock and ICU-Septic shock, respectively. Statistical analyses were performed using the Kruskal–Wallis test **(A, B)**.

Supplementary Figure 2 (related to **Figure 1**). PCA analysis of transcriptome sequencing features in septic shock patients. **(A)** PCA of peripheral blood neutrophils from healthy donors and septic patients across disease states.

Supplementary Figure 3 (related to **Figure 1**). Flow cytometry gate strategy for neutrophils in human peripheral blood. **(A)**Gating strategy for flow cytometry analysis.

Supplementary Figure 4 (related to **Figure 2**).Differences in various subsets of neutrophils. **(A)** Violin plots showing pseudotime of neutrophils in human PBs across subsets, tested with student t-test. *P < 0.05, **P < 0.01, ***P < 0.001.

Supplementary Figure 5 (related to **Figure 3**). Differences in gene expression of neutrophils between healthy donors and septic patients Differences in various subsets of neutrophils. **(A)** Neutrophil gene expression in septic patients at varying stages displays noteworthy differences. Only genes with counts > 1 are presented above. **(B-D)** Heatmaps displaying the expression patterns of selected genes related to the IL-1 receptor **(B)**, MMP **(C)**, and TLR **(D)**.

Supplementary Figure 6 (related to **Figure 6**). Neutrophils generate soluble CD121 in patients with septic shock. **(A)** Representative flow cytometry analysis of the frequencies of CD121b^+^ neutrophils in healthy donor (middle panel) and septic shock patient (lower panel). **(B)** Representative flow cytometry analysis of the frequencies of CD121b^+^ T cells in healthy donor (middle panel) and septic shock patient (lower panel). **(C)** Soluble IL1R2 (sCD121b) concentrations secreted by peripheral blood neutrophils from healthy donors (n=5) and patients with septic shock (n=5) after 6 h or 18 h of *in vitro* culture. Statistical analyses were performed using two-tailed unpaired Student’s t test (C) and Mann–Whitney test (C).

Supplementary Table 2 Key resources table.
