# Supplementary figures and images for: CD121b-positive neutrophils predict immunosuppression in septic shock
Source: Front Immunol. 2025 Mar 31;16:1565797. doi: 10.3389/fimmu.2025.1565797 (PMC11994419; doi:10.3389/fimmu.2025.1565797)

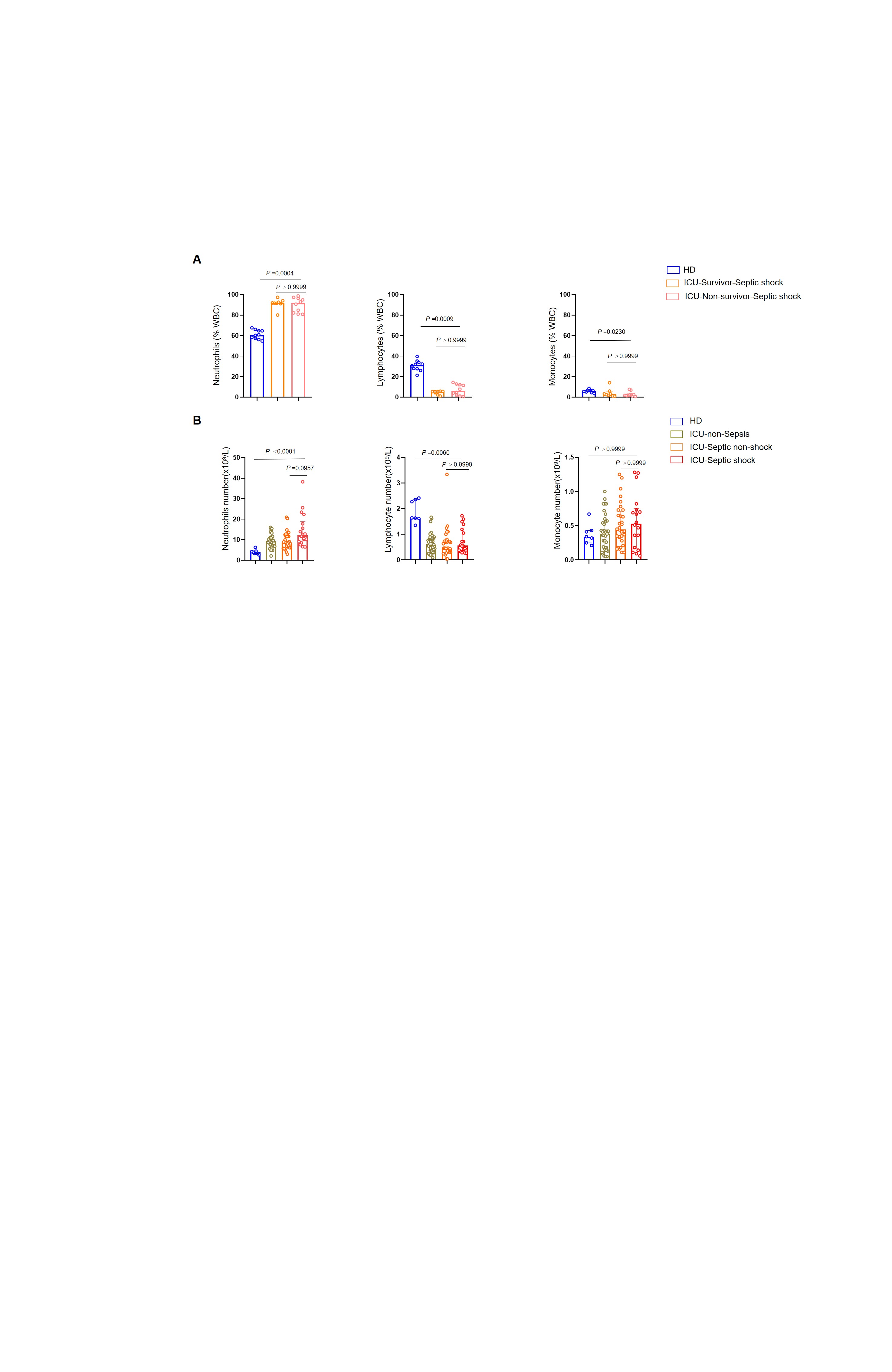

Supplement: Supplementary Figure 1 — (related to Figure 1 ). Increased proportion and absolute number of neutrophils in patients with septic shock. (A) Proportion neutrophils, lymphocytes, and monocytes in peripheral blood of HDs, patients surviving from septic shock (ICU-Survivor-Septic shock) and patients with septic shock leading to fatal outcome (ICU-Non-Survivor-Septic shock). n = 10, 8 and 10 for HD, ICU-Survivor-sepsis and ICU-Non-survivor-sepsis, respectively. (B) Cell count of neutrophils, lymphocytes, and monocytes in peripheral blood of HDs, non-sepsis ICU patients (ICU-non-Sepsis), patients without septic shock(ICU-Septic non-shock) and patients with septic shock (and ICU-Septic shock). n = 7, 37, 32 and 18 for HD, ICU-non-sepsis, ICU-Septic non-shock and ICU-Septic shock, respectively. Statistical analyses were performed using the Kruskal–Wallis test (A, B). [file Image1.jpeg]

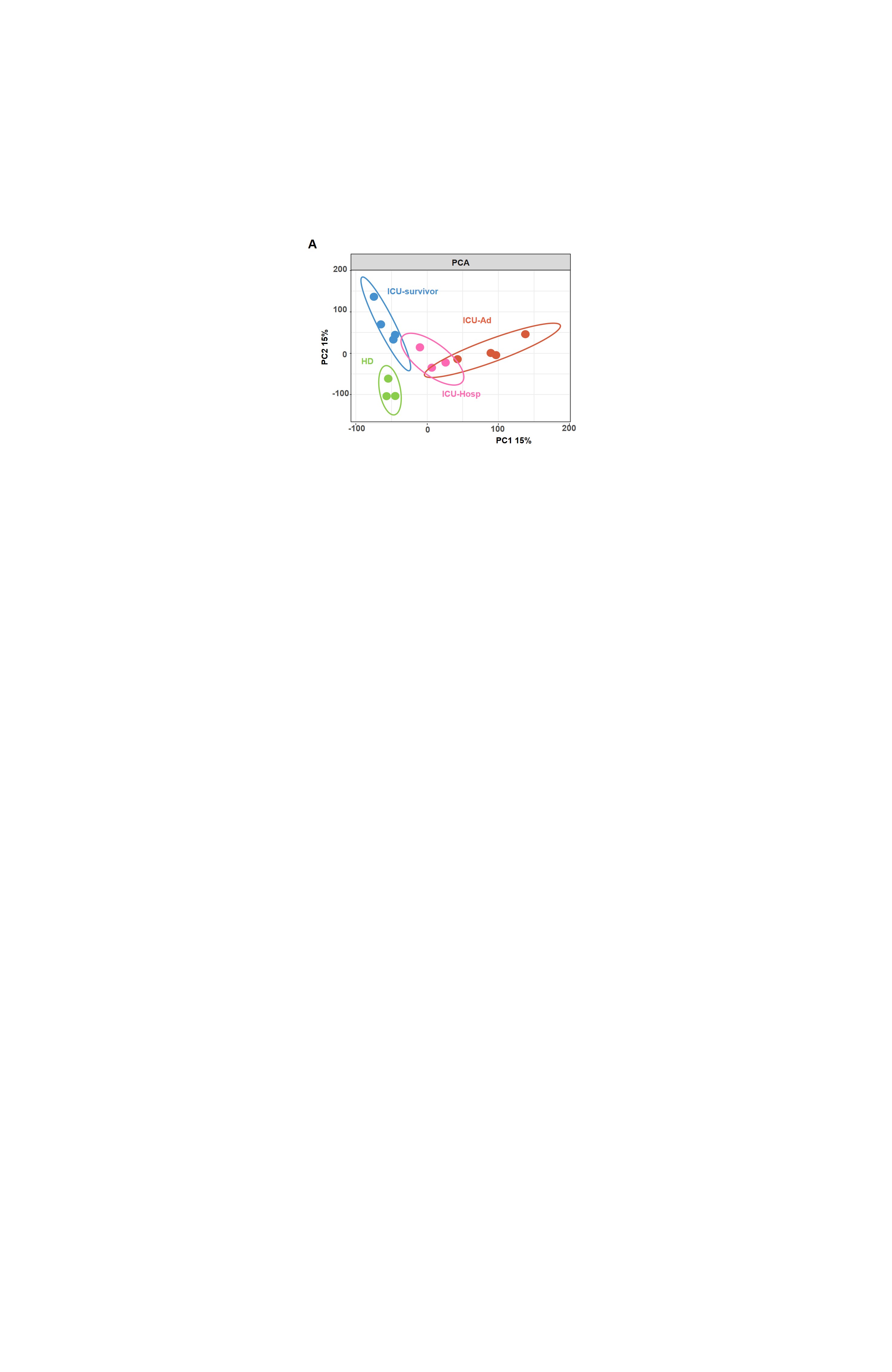

Supplement: Supplementary Figure 2 — (related to Figure 1 ). PCA analysis of transcriptome sequencing features in septic shock patients. (A) PCA of peripheral blood neutrophils from healthy donors and septic patients across disease states. [file Image2.jpeg]

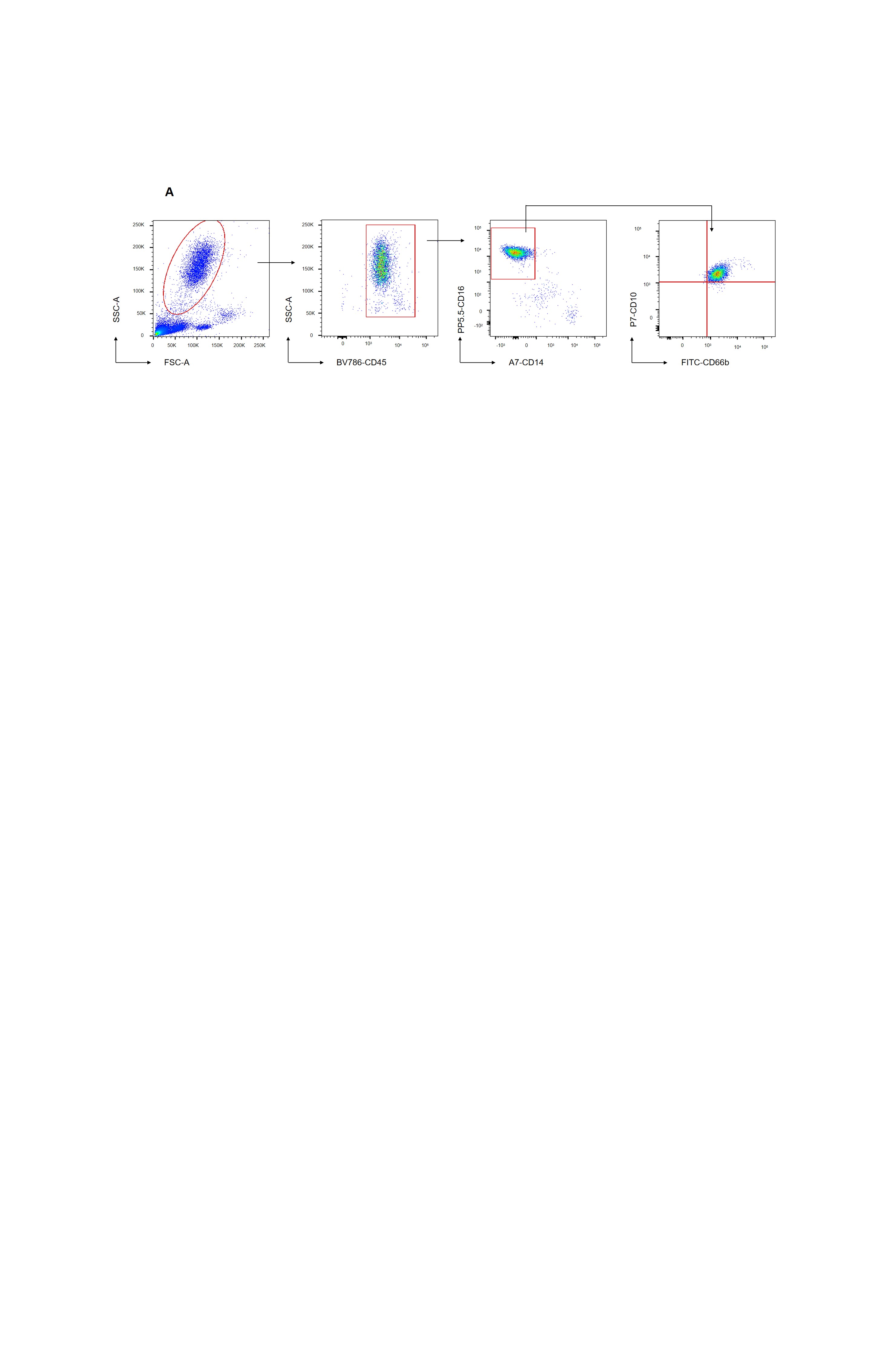

Supplement: Supplementary Figure 3 — (related to Figure 1 ). Flow cytometry gate strategy for neutrophils in human peripheral blood. (A)Gating strategy for flow cytometry analysis. [file Image3.jpeg]

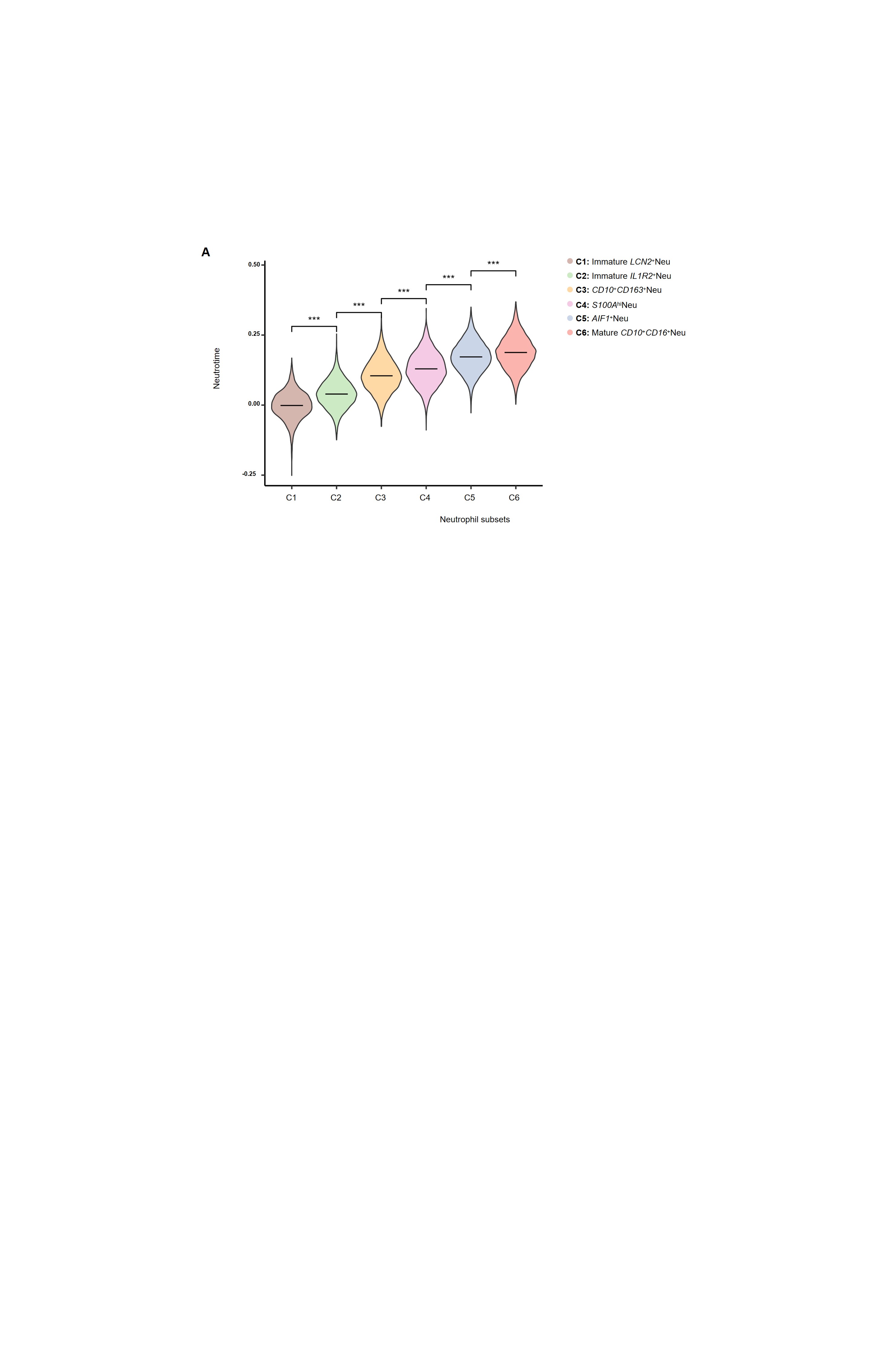

Supplement: Supplementary Figure 4 — (related to Figure 2 ).Differences in various subsets of neutrophils. (A) Violin plots showing pseudotime of neutrophils in human PBs across subsets, tested with student t-test. *P < 0.05, **P < 0.01, ***P < 0.001. [file Image4.jpeg]

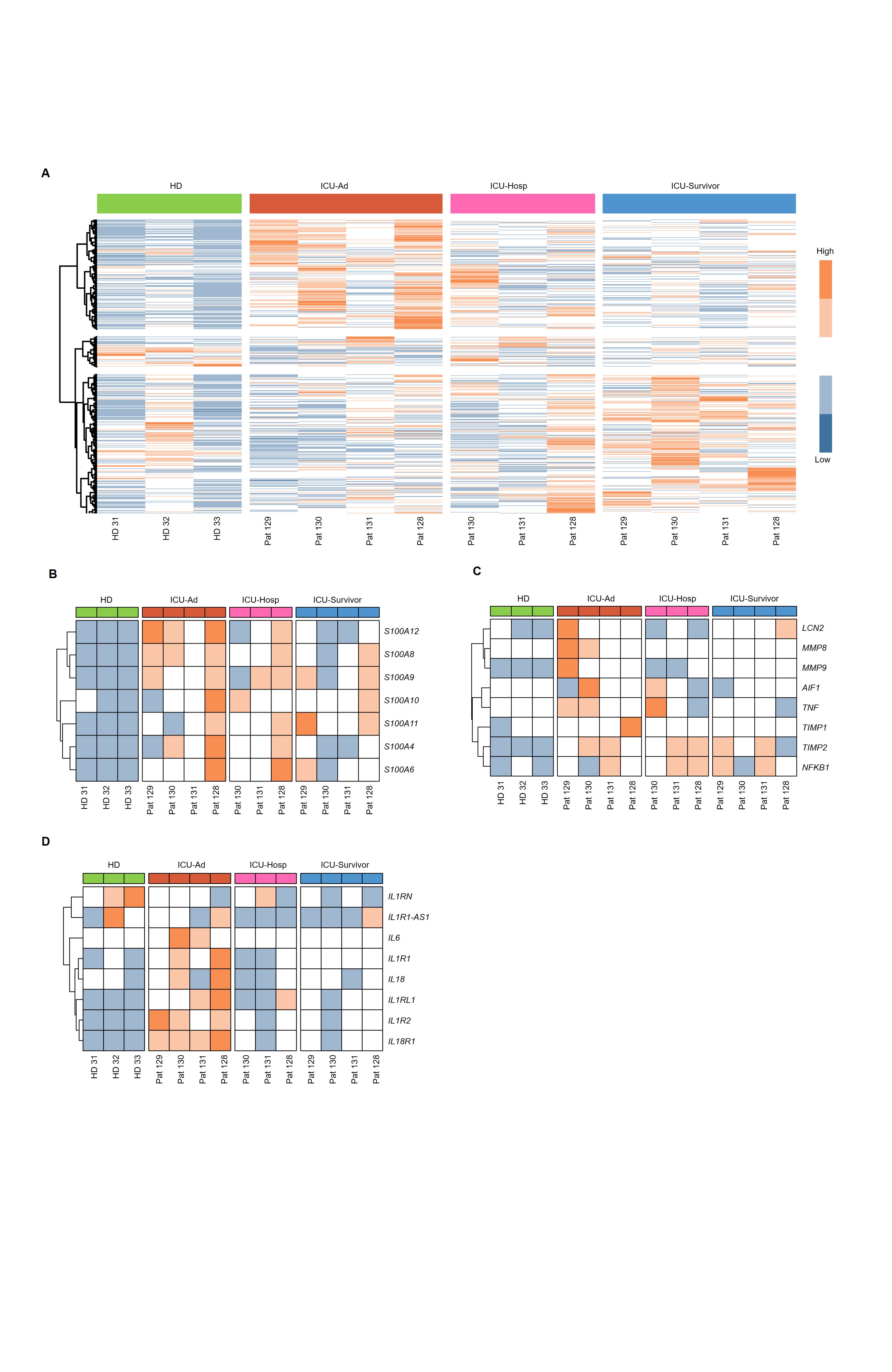

Supplement: Supplementary Figure 5 — (related to Figure 3 ). Differences in gene expression of neutrophils between healthy donors and septic patients Differences in various subsets of neutrophils. (A) Neutrophil gene expression in septic patients at varying stages displays noteworthy differences. Only genes with counts > 1 are presented above. (B-D) Heatmaps displaying the expression patterns of selected genes related to the IL-1 receptor (B), MMP (C), and TLR (D). [file Image5.jpeg]

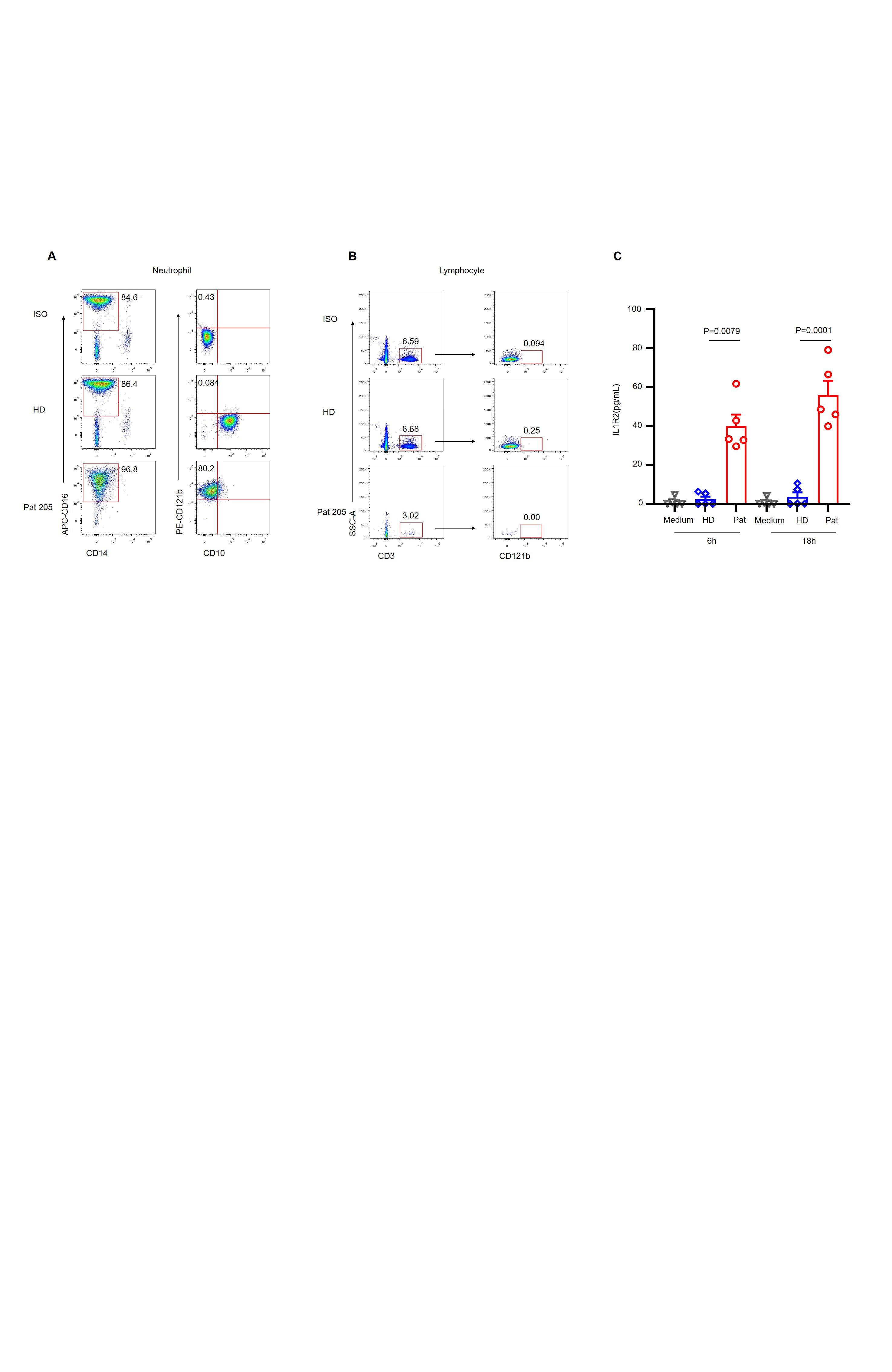

Supplement: Supplementary Figure 6 — (related to Figure 6 ). Neutrophils generate soluble CD121 in patients with septic shock. (A) Representative flow cytometry analysis of the frequencies of CD121b+ neutrophils in healthy donor (middle panel) and septic shock patient (lower panel). (B) Representative flow cytometry analysis of the frequencies of CD121b+ T cells in healthy donor (middle panel) and septic shock patient (lower panel). (C) Soluble IL1R2 (sCD121b) concentrations secreted by peripheral blood neutrophils from healthy donors (n=5) and patients with septic shock (n=5) after 6 h or 18 h of in vitro culture. Statistical analyses were performed using two-tailed unpaired Student’s t test (C) and Mann–Whitney test (C). [file Image6.jpeg]
