# Supplementary material for: CD121b-positive neutrophils predict immunosuppression in septic shock
Source: Front Immunol. 2025 Mar 31;16:1565797. doi: 10.3389/fimmu.2025.1565797 (PMC11994419; doi:10.3389/fimmu.2025.1565797)
Supplement: Supplementary file 7 [file Table1.docx]

**Key resources table**

| REAGENT or RESOURCE | SOURCE | IDENTIFIER |
| --- | --- | --- |
| Antibodies | | |
| Anti-Human FITC-CD11b, clone ICRF44 | BD | Cat # 557701, RRID: [AB_2129268](https://www.antibodyregistry.org/AB_2129268) |
| Anti-Human FITC-CD66b, clone G10F5 | BD | Cat # 555724, RRID: [AB_396067](https://www.antibodyregistry.org/AB_396067) |
| Human IL-1 RII PE-conjugated Antibody, clone 34141 | R&D Systems | Cat # FAB663P, RRID: [AB_1964613](https://www.antibodyregistry.org/AB_1964613) |
| Anti-human PerCP/Cyanine5.5-CD45, clone 2D1 | BioLegend | Cat # 368504, RRID: [AB_2566352](https://www.antibodyregistry.org/AB_2566352) |
| Anti-human PerCP/Cyanine5.5-CD16, clone 3G8 | BioLegend | Cat # 302028, RRID: [AB_893262](https://www.antibodyregistry.org/AB_893262) |
| Anti-Human PE-Cy7-CD10, clone HI10a | BD | Cat # 565282, RRID: [AB_2739153](https://www.antibodyregistry.org/AB_2739153) |
| Anti-Human APC-Cy7-CD14, clone MφP9 | BD | Cat # 557831, RRID: [AB_396889](https://www.antibodyregistry.org/AB_396889) |
| Anti-Human BV786-CD45, clone HI30 | BD | Cat # 563716, RRID: [AB_2716864](https://www.antibodyregistry.org/AB_2716864) |
| Anti-human BV510-CD62L, clone DREG-56 | BioLegend | Cat # 304844, RRID: [AB_2617003](https://www.antibodyregistry.org/AB_2617003) |
| Anti-human BV605-CD63, clone H5C6 | BioLegend | Cat # 353048, RRID: [AB_2888782](https://www.antibodyregistry.org/AB_2888782) |
| Anti-human APC-CD11b, clone ICRF44 | BioLegend | Cat # 301310, RRID: [AB_314162](https://www.antibodyregistry.org/AB_314162) |
| Anti-human APC-CD16, clone 3G8 | BioLegend | Cat # 302012 , RRID: [AB_314211](https://www.antibodyregistry.org/AB_314211) |
| Human IL-1RII Antibody | R&D Systems | Cat# AF-263-NA, RRID: [AB_354431](https://www.antibodyregistry.org/AB_354431) |
| Biological samples | | |
| Peripheral blood of healthy controls and sepsis patients | The First Affiliated Hospital of the University of Science and Technology of China | Ethics Number: 2022KY-No.158. |
| Chemicals, peptides, and recombinant proteins | | |
| Recombinant Human IL-1 RII | R&D Systems | Cat # 263-2R |
| All-trans Retinoic Acid | Selleck | Cat # S1653 |
| Dexamethasone | Sangon Biotech | Cat # A601187-0005 |
| L-Arginine | Sigma | Cat # A8094-25G |
| LPS | Solarbio | Cat # L8880 |
| Human Granulocyte Colony Stimulating Factor Injection | HANGZHOU JIUYUAN GENE ENGINEERING Co. LTD | S10980030 |
| Critical commercial assays | | |
| Human IL-10 ELISA Kit | MultiSciences | Cat # EK110 |
| Human IL-1β ELISA | MultiSciences | Cat # EK101 |
| Human IL-6 ELISA | MultiSciences | Cat # EK106 |
| Human IL-8 ELISA Kit | MultiSciences | Cat # EK108 |
| Human IL-4 ELISA Kit | MultiSciences | Cat # EK104 |
| Human IL-1 RII Quantikine ELISA Kit | R&D Systems | Cat # DR1B00 |
| Human IL-1ra/IL-1F3 ELISA Kit | MultiSciences | Cat # EK1132 |
| Human Lipocalin-2/NGAL Quantikine ELISA Kit | R&D Systems | Cat # DLCN20 |
| Deposited data | | |
| scRNA sequencing data | This paper | GSA: HRA007054  GSA: HRA007060 |
| Bulk-RNA sequencing data | This paper | GSA: HRA007050 |
| Experimental models: Organisms/strains | | |
| Mouse: NOD/ShiLtJGpt-Prkdcem26Cd52Il2rgem26Cd22/Gpt (NCG) | GemPharmatech | Strain NO. T001475 |
| Oligonucleotides | | |
| qPCR primers for various genes | This paper | Table 2 |
| Software and algorithms | | |
| FlowJo software version 10.6.2 | FlowJo, LLC | https://www.flowjo.com/ |
| Prism version 8 | Graphpad | https://www.graphpad.com/ |
